# Towards a System Level Understanding of Non-Model Organisms Sampled from the Environment: A Network Biology Approach

**DOI:** 10.1371/journal.pcbi.1002126

**Published:** 2011-08-25

**Authors:** Tim D. Williams, Nil Turan, Amer M. Diab, Huifeng Wu, Carolynn Mackenzie, Katie L. Bartie, Olga Hrydziuszko, Brett P. Lyons, Grant D. Stentiford, John M. Herbert, Joseph K. Abraham, Ioanna Katsiadaki, Michael J. Leaver, John B. Taggart, Stephen G. George, Mark R. Viant, Kevin J. Chipman, Francesco Falciani

**Affiliations:** 1 School of Biosciences, The University of Birmingham, Birmingham, United Kingdom; 2 Institute of Aquaculture, University of Stirling, Stirling, Scotland, United Kingdom; 3 Yantai Institute of Coastal Zone Research, Academy of Sciences, Yantai, PR. China; 4 Cefas, Weymouth Laboratory, Weymouth, Dorset, United Kingdom; 5 Department of Epidemiology and Biostatistics, Case Western Reserve University, Cleveland, Ohio, United States of America; 6 Department of Animal Science, Iowa State University, Ames, Iowa, United States of America; University of Zurich and Swiss Institute of Bioinformatics, Switzerland

## Abstract

The acquisition and analysis of datasets including multi-level *omics* and physiology from non-model species, sampled from field populations, is a formidable challenge, which so far has prevented the application of systems biology approaches. If successful, these could contribute enormously to improving our understanding of how populations of living organisms adapt to environmental stressors relating to, for example, pollution and climate. Here we describe the first application of a network inference approach integrating transcriptional, metabolic and phenotypic information representative of wild populations of the European flounder fish, sampled at seven estuarine locations in northern Europe with different degrees and profiles of chemical contaminants. We identified network modules, whose activity was predictive of environmental exposure and represented a link between molecular and morphometric indices. These sub-networks represented both known and candidate novel adverse outcome pathways representative of several aspects of human liver pathophysiology such as liver hyperplasia, fibrosis, and hepatocellular carcinoma. At the molecular level these pathways were linked to TNF alpha, TGF beta, PDGF, AGT and VEGF signalling. More generally, this pioneering study has important implications as it can be applied to model molecular mechanisms of compensatory adaptation to a wide range of scenarios in wild populations.

## Author Summary

Understanding how living organisms adapt to changes in their natural habitats is of paramount importance particularly in respect to environmental stressors, such as pollution or climate. Computational models integrating the multi-level molecular responses with organism physiology are likely to be indispensable tools to address this challenge. However, because of the difficulties in acquiring and integrating data from non-model species and because of the intrinsic complexity of field studies, such an approach has not yet been attempted. Here we describe the first example of a global network reconstruction linking transcriptional and metabolic responses to physiology in the flatfish, European flounder, a species currently used to monitor coastal waters around Northern Europe. The model we developed has revealed a remarkable similarity between network modules predictive of chemical exposure in the environment and pathways involved in relevant aspects of human pathophysiology. Generally, the approach we have pioneered has important implications as it can be applied to model molecular mechanisms of compensatory adaptation to a wide range of scenarios in wild populations.

## Introduction

Modelling the responses and compensatory adaptations of living organisms to a changing environment is extremely important both in terms of scientific understanding and for its potential impact on global health. Although computational modelling of ecological systems has been utilised in ecotoxicology, the application of systems biology approaches to non-model organisms in general presents formidable difficulties, partly due to limited sequence information for environmentally relevant sentinel species. Moreover, the number of samples and the depth of information available are often limited and there may be a lack of truly relevant physiological endpoints. Thus, omics have proven effective in finding responses of aquatic organisms to model toxicants in laboratory-based experiments [1] but the environment poses a greater challenge as anthropogenic contaminants are present as complex mixtures and responses will additionally be dependent upon natural life history traits and other environmental factors.

Relatively few omics studies have focussed upon the ecotoxicology of environmentally sampled fish [2]–[7]. Although we have previously shown [8], [9] that expression of stress response genes could be used to distinguish fish from environmental sampling sites with different underlying contaminant burdens, this gave little insight to the health outcomes of these molecular differences. In this context, identifying molecular mechanisms of compensatory and toxic responses from observational data (reverse engineering), an approach that has been so successful in clinical studies and in laboratory model organisms, is highly challenging in field studies. We addressed this challenge by developing a novel network inference strategy based on the integration of multi-level measurements of populations of fish exposed to a diverse spectrum of environmental pollutants. This provides a useful model for a network biology approach generally applicable to non-model species and represents a breakthrough in the way we study the mechanisms whereby organisms respond to chemical exposure in the environment. We directed our efforts towards modelling molecular networks representative of populations of the flatfish European flounder (*Platichthys flesus*) sampled from marine environments of North Western Europe, including locations significantly impacted by anthropogenic chemical contaminants.

The study integrated measurements representing a broad spectrum of samples characterized using transcriptomics, metabolomics, conventional biomarkers and analysis of chemicals in sediments from the sampling sites. Previous studies have shown both anthropogenic contamination and higher prevalence of pre-neoplastic and neoplastic lesions in flounder from the Elbe estuary [10] and from the Mersey and Tyne [11], together with elevated levels of hepatic DNA adducts at these sites [12]. Data integration was achieved by implementing a systems biology framework for network reconstruction, starting from cross-species mapping of sequence information to the integration of multi-level datasets within a framework for network inference [13] and culminating in the identification of network modules predictive of physiological responses to chemical exposure, valuable for marine monitoring [14].

The networks we identified demonstrate a remarkable parallel between human liver carcinogenesis and environmental effects on fish liver as well as revealing potentially novel adaptation mechanisms. The broader application of network biology approaches to other non-model species sampled from the environment is therefore likely to profoundly change our understanding of how living systems are likely to adapt to complex environments.

## Results

### The molecular state of flounder liver reflects chemical exposure

An important assumption in many eco-toxicology studies is that the molecular states of organisms reflect their biological responses to complex chemical mixtures present within that environment. Indirect evidence suggests that this hypothesis may be correct. For example, consistent with previous studies [9], we have identified genes and metabolites that were differentially expressed between environmental sites (the results obtained are shown in detail in Table 1 and Text S1, Tables S1 and S2). Many of these were either known to be associated with stress responses or were previously shown to respond to anthropogenic chemical contaminants in fish. Although these results were encouraging they did not provide a direct link between molecular status and response to specific chemicals. Since sediment chemistry data was available, we assessed whether chemical contaminant profiles could be inferred from gene expression data and whether these would at least partially match the known sediment composition. Our analysis was performed by linking genes differentially expressed between each sampling site and the reference site, with chemical-gene relationships within the Comparative Toxicology Database (CTD) [15]. The Alde estuary was chosen as the reference site due to its low concentrations of major anthropogenic chemical contaminants (Table 1), both in sediment and in flounder livers. These significant associations may be regarded as predictive of the most important classes of chemicals exerting their biological effects upon flounder gene expression amongst the highly complex chemical mixtures within the sediments at these sites. Results were consistent with the initial hypothesis, as where contaminants were highlighted both by chemistry and CTD analysis; sediment concentrations all exceeded the lower OSPAR ecotoxicological assessment criteria, except for PAHs at the Morecambe site. At Brunsbuttel, elevated chromium and polychlorinated biphenyls (PCBs); at Cuxhaven, chromium, nickel, lead, zinc, polycyclic aromatic hydrocarbons (PAHs) and PCBs; at Helgoland nickel, zinc, manganese and PCBs, at Mersey PCBs; at Morecambe Bay arsenic, nickel and PAHs; and at Tyne arsenic and PCBs were all predicted by the CTD analyses and confirmed by chemistry data (Table 2 and Table S3). PAHs were predicted at Morecambe and Cuxhaven, with the AhR-inducer beta-naphthoflavone predicted at Brunsbuttel, Helgoland and Tyne, consistent with our finding of CYP1A transcriptional induction at all sites in comparison with the Alde. Additionally, Ingenuity Pathway Analysis (IPA) of all genes identified as significantly differentially expressed between sites showed significant associations with a number of toxicologically important processes and outcomes (Table 3).

**Table 1:** Chemistry, fish morphology, histopathology and protein biomarkers.

A. Fish Measurements	Alde	Tyne	Mersey	Morecambe	Brunsbuttel	Helgoland	Cuxhaven
Length (cm) *	**23.3+/−3.13**	18.56+/−4.8	**27.72+/−4.75**	**28.66+/−2.74**	15.98+/−1.7	**21.45+/−3.52**	**17.58+/−3.12**
Weight (g) *	**127.65+/−56.32**	90.33+/−76.31	**181.33+/−83.61**	**196.41+/−42.42**	73.16+/−22.23	**159.78+/−64.63**	**106+/−51.06**
Condition Factor K *	**0.96+/−0.08**	**1.19+/−0.22**	0.79+/−0.07	0.82+/−0.07	**1.74+/−0.14**	**1.56+/−0.22**	**1.82+/−0.14**
Liver weight (g) *	**1.63+/−0.65**	1.53+/−1.33	**2.49+/−1.18**	**2.76+/−0.98**	1.05+/−0.38	**1.62+/−0.82**	1.15+/−0.75
HSI *	**1.39+/−0.4**	**1.7+/−0.93**	**1.4+/−0.39**	**1.42+/−0.42**	**1.45+/−0.35**	1.01+/−0.3	1.1+/−0.48
Gonad weight (g) *	nd	nd	nd	nd	0.15+/−0.08	**0.77+/−0.75**	**1.4+/−1.59**
GSI *	nd	nd	nd	nd	0.21+/−0.1	**0.45+/−0.4**	**1.26+/−1.37**

B. Sediment Metals	Alde	Tyne	Mersey	Morecambe	Brunsbuttel	Helgoland	Cuxhaven
Al (%)	nd	5.81	3.90	1.62	2.8	5.1	2.6
As (mg/kg)	16.00	14.48	11.60	9.74	21.9	33	18.8
Cd (mg/kg)	<0.02	**1.72**	0.49	0.08	0.4	0.9	0.44
Cr (mg/kg)	7.00	71.35	66.28	26.60	59.4	81.8	76.4
Cu (mg/kg)	5.00	48.75	26.20	23.70	25.7	26	19.7
Fe (%)	nd	3.33	2.43	4.12	3.1	4.8	2.9
Hg (mg/kg)	<0.01	0.24	**0.67**	0.02	**0.84**	0.47	**0.78**
Li (mg/kg)	nd	63.98	34.80	16.60	36.3	57.4	30.1
Mn (mg/kg)	nd	575.80	826.40	350.00	2009	1166	1388
Ni (mg/kg)	9.00	36.58	22.92	12.47	26	35.7	24.6
Pb (mg/kg)	29.00	**248.20**	**50.80**	15.00	**85**	50	32.5
Zn (mg/kg)	63.00	**536.40**	179.80	33.30	231	230	188

C. Fish Liver Metals(mg/kg wet wt)	Alde	Tyne	Mersey	Morecambe	Brunsbuttel	Helgoland	Cuxhaven
As	7.5	2	12	nd	3.98	1.65	0.68
Cd	0.31	0	0.19	0.161	0.1	0.07	0.16
Cr	0	0.23	0	nd	1.2	0.35	0.33
Cu	26	8	27	nd	nd	24.9	nd
Fe	262	239	220	nd	nd	nd	nd
Hg	0.1	0.02	0.73	0.009	nd	0.05	nd
Ni	0.17	0.33	0.2	nd	nd	nd	nd
Pb	0	0.35	0.94	0.53	0.1	0.34	0.1
Se	2.6	2	2.5	nd	nd	nd	nd
Zn	59	46	57	nd	nd	nd	nd

D. Sediment PAHs (mg/kg dry wt)	Alde	Tyne	Mersey	Morecambe	Brunsbuttel	Helgoland	Cuxhaven
Anthracene	1.4	354.0	420.4	<10	82.0	123.0	80.0
Benzo[a]anthracene	6.0	708.8	880.4	3.1	553.0	31.0	196.0
Benzo[a]pyrene	4.2	735.8	**1441.0**	2.3	380.0	39.0	14.0
Benzo[ghi]perylene	5.3	516.4	**1282.8**	<10	392.0	57.0	310.0
Chrysene/Triphenylene	3.3	**1484.0**	**1001.0**	3.6	992.0	30.0	51.0
Fluoranthrene	1.5	**7192.5**	3163.8	4.2	1044.0	59.0	77.0
Indeno[123-cd]pyrene	4.3	381.2	919.2	<10	404.0	61.0	35.0
Napthalene	6.0	122.2	nd	18.0	187.0	30.0	7.0
Phenanthrene	10.0	**5590.0**	**1533.0**	11.0	566.0	42.0	8.0
Pyrene	7.4	**6477.4**	**3355.2**	3.8	**625.0**	51.1	5.6
Sum of Sediment PAHs	49.4	23562.3	13996.8	<76	5225.0	523.1	783.6

E. Fish Liver PAHs (mg/kg wet wt)	Alde	Tyne	Mersey	Morecambe	Brunsbuttel	Helgoland	Cuxhaven
Acenaphthylene	nd	12.7	4.9	nd	nd	nd	nd
acenaphthene	1.59	150.7	17.5	nd	nd	nd	nd
Anthracene	nd	13.5	4.3	nd	nd	nd	nd
benzo[a]anthracene	nd	12.0	1.7	nd	nd	nd	nd
C1-Napthalene	11.41	144.6	102.7	nd	nd	nd	nd
C1-Phenanthrene/anthracene	nd	79.1	0.0	nd	nd	nd	nd
C2N-Napthalene	30.22	217.8	221.8	nd	nd	nd	nd
C3N-Napthalene	31.97	292.0	308.3	nd	nd	nd	nd
Chrysene	nd	7.3	8.8	nd	nd	nd	nd
Fluoroanthrene	1.65	43.0	4.6	nd	nd	nd	nd
Fluorene	3.63	49.0	13.6	nd	nd	nd	nd
Napthalene	1.23	108.0	11.0	nd	nd	nd	nd
Phenanthrene	5.07	92.0	12.0	nd	nd	nd	nd
Pyrene	nd	20.0	4.5	nd	nd	nd	nd
Sum of PAHs	86.76	1241.77	715.71	nd	nd	nd	nd

F. Sediment PCBs (mg/kg dry wt)	Alde	Tyne	Mersey	Morecambe	Brunsbuttel	Helgoland	Cuxhaven
CB28	0.02	1.23	9.17	<0.1	<0.7	<0.2	<0.6
CB52	0.01	0.95	3.72	<0.1	0.95	0.3	<0.4
CB101	nd	1.04	7.06	<0.1	0.80	0.4	<0.6
CB118	0.00	1.56	**11.42**	<0.1	nd	0.5	<0.1
CB138	0.01	2.38	**13.04**	<0.1	1.55	0.3	1.06
CB153	0.01	1.66	**10.30**	<0.1	1.55	0.2	0.99
CB180	0.00	0.60	2.04	<0.1	<0.75	1	0.63
Sum of ICES 7 Sediment PCBs	0.05	9.41	**56.75**	<0.7	4.95	2.7	2.68
Organic Carbon (%)	nd	4.48	1.56	0.1	2.90	1.9	2.90

G. Fish Liver PCBs (mg/kg wet wt)	Alde	Tyne	Mersey	Morecambe	Brunsbuttel	Helgoland	Cuxhaven
CB28	0.016	nd	0.015	0.005	0.037	0.005	0.021
CB52	0.013	0.009	0.021	0.007	0.066	0.004	0.051
CB101	nd	nd	0.11	0.011	0.037	0.008	0.009
CB118	0.004	0.011	0.033	0.01	0.081	0.007	0.007
CB138	0.009	0.019	0.045	0.02	0.257	0.01	0.01
CB153	0.011	0.025	0.065	0.024	0.374	0.18	0.291
CB180	0.004	0.015	0.027	0.01	0.165	0.01	0.241
Sum of ICES 7 Fish liver PCBs	0.057	0.079	0.316	0.090	1.017	0.224	0.630

H. Histopathology	Alde	Tyne	Mersey	Morecambe	Brunsbuttel	Helgoland	Cuxhaven
Lymphocystis (%)	0	0	0	8	nd	nd	nd
Skin ulcer (%)	5	0	0	0	nd	nd	nd
Liver nodules (%)	0	0	6	0	nd	nd	nd
Multiple liver nodules (%)	0	0	6	0	nd	nd	nd
*Lepeophtheirus* sp. (%)	75	13	56	88	nd	nd	nd
*Acanthochondria* sp. (%)	70	6	56	79	nd	nd	nd
*Anisakidae* sp. (Rubin 1) (%)	5	44	28	4	nd	nd	nd
*Glugea* sp. (%)	5	0	6	8	nd	nd	nd
Zonal differences on liver (%)	0	6	6	0	nd	nd	nd
Phospholipoidosis (%)	0	6	28	4	nd	nd	nd
Fibrillar Inclusions (%)	0	31	50	4	nd	nd	nd
H&N pleomorphism (%)	0	0	28	0	nd	nd	nd
Hydropic degeneration (%)	0	0	6	4	nd	nd	nd
Clear-cell FCA (%)	0	0	6	0	nd	nd	nd
Vacuolated FCA (%)	5	0	11	0	nd	nd	nd
Eosinophillic FCA (%)	5	0	28	21	nd	nd	nd
Basophillic FCA (%)	0	0	6	0	nd	nd	nd
Hepatocellular adenoma (%)	0	0	6	0	nd	nd	nd
Hepatocellular carcinoma (%)	0	0	11	0	nd	nd	nd
Coagulative necrosis (%)	10	19	17	8	nd	nd	nd
Apoptosis (%)	0	0	6	0	nd	nd	nd
Lipoidosis (%)	10	0	11	0	nd	nd	nd
Melanomacrophage centre (%)	10	19	67	54	nd	nd	nd
Lymphocytic/monocytic infiltration (%)	10	0	28	21	nd	nd	nd
Granuloma (%)	0	6	0	0	nd	nd	nd
Regeneration (%)	5	19	22	13	nd	nd	nd
Any liver abnormalites detected (%)	40	75	89	67	nd	nd	nd

I. Biomarkers	Alde	Tyne	Mersey	Morecambe	Brunsbuttel	Helgoland	Cuxhaven
VTG (mg/ml)	0.03+/−0.04	0.07+/−0.11	87.55+/−369.97	**47.01+/−230.24**	**0.16+/−0.13**	nd	nd
GR (nmol/mg) *	29.18+/−25.66	**38.29+/−9.31**	21.98+/−10.46	18.03+/−6.19	18.8+/−11.45	nd	nd
GST (umol/mg) *	**1.15+/−0.45**	**1.62+/−0.67**	0.98+/−0.39	0.78+/−0.29	**1.8+/−1.56**	nd	nd
EROD (pmol/mg) *	21.6+/−17.3	161.37+/−230.29	**40.01+/−30.39**	**51.4+/−49.93**	**80.64+/−98.3**	nd	nd
MT (ug/mg) *	2.49+/−1.52	3.1+/−1.36	**5.6+/−3.42**	2.24+/−2.15	**8.85+/−7.41**	nd	nd

Table 1 A,I bold = significant by T-test P<0.05 versus lowest value, data shown as mean +/− SD,

*significant by ANOVA at P<0.05.

Table 1 B,D,F underlined = >lower OSPAR EAC, bold underlined >higher OSPAR EAC. nd = not determined.

**Table 2:** Environmental contaminants predicted from CTD.

Brunsbuttel	FDR	Mersey	FDR
Perfluorooctane sulfonic acid*	4.02E-03	Lindane	6.79E-02
Systhane	6.42E-03	Systhane	6.79E-02
Chlorine	6.92E-03	Fluconazole	7.15E-02
Endosulfan*	1.15E-02	Phenobarbital	7.15E-02
Potassium dichromate*	2.60E-02	Dimethyl sulfoxide	7.36E-02
2,4,5,2′,4′,5′-Hexachlorobiphenyl*	3.90E-02	Flavonoids	7.36E-02
Chromium*	3.92E-02	Polyphenols	7.49E-02
Polychlorinated biphenyls*	3.92E-02	Tobacco smoke pollution	7.89E-02
Dieldrin*	9.57E-02	Ethinyl-estradiol*	8.68E-02
Ethinyl-estradiol*	9.90E-02	2,4,5,2′,4′,5′-Hexachlorobiphenyl*	9.80E-02

Cuxhaven	FDR	Morecambe Bay	FDR
Melatonin	2.52E-02	Paraquat	1.12E-03
beta-Naphthoflavone	2.54E-02	Astemizole	6.81E-02
Catechin	2.54E-02	Sodium arsenite*	6.81E-02
Chromium*	3.10E-02	Cycloheximide	7.13E-02
Epicatechin gallate	3.65E-02	Propiconazole	7.13E-02
Nickel sulfate*	4.77E-02	Isoflavones	7.20E-02
Lead*	6.93E-02	Corticosterone	7.42E-02
Zinc*	6.93E-02	Estradiol*	7.42E-02
Benzo(a)pyrene-7,8-dione*	6.93E-02	Benzo(a)pyrene*	8.16E-02
Polychlorinated biphenyls*	8.30E-02	Nickel chloride*	9.45E-02

Helgoland	FDR	Tyne	FDR
Epicatechin gallate	3.78E-02	Melatonin	1.30E-02
Gallocatechol	3.78E-02	Paraquat	3.32E-02
Permethrin	3.78E-02	Epigallocatechin gallate	4.03E-02
Ethinyl Estradiol	4.08E-02	Astemizole	4.82E-02
Astemizole	4.39E-02	Catechin	4.97E-02
Melatonin	4.48E-02	Dieldrin	5.11E-02
Zinc*	4.57E-02	Epicatechin gallate	5.64E-02
Nickel sulfate*	6.60E-02	Estrone	5.64E-02
Polychlorinated biphenyls*	7.15E-02	Sodium arsenite*	8.18E-02
Manganese*	9.73E-02	Polychlorinated biphenyls*	8.82E-02

CTD gene-chemical interactions statistically significantly (FDR<0.1) associated with gene expression changes in flounders from each sampling site in comparison with Alde fish. The most significant associations are shown together with those supported by chemistry and other data (starred), full data are shown in Table S3.

**Table 3:** Ingenuity annotations associated with site and histopathological differences.

Canonical Pathways	FDR	Associations (FDR<0.05)
Protein Ubiquitination Pathway	3.55E-04	
Glutathione Metabolism	5.66E-04	
NRF2-mediated Oxidative Stress Response	6.73E-04	Phospholipidosis
Lysine Degradation	8.01E-04	Intersex
Acute Phase Response Signaling	1.77E-03	Vacuolar FCA
Granzyme B Signaling	1.03E-02	
Glycine, Serine and Threonine Metabolism	2.00E-02	
Methane Metabolism	2.10E-02	
Glycolysis/Gluconeogenesis	2.01E-02	
Histidine Metabolism	2.01E-02	
Pyruvate Metabolism	2.01E-02	
Metabolism of Xenobiotics by Cytochrome P450	2.36E-02	Phospholipidosis
Regulation of eIF4 and p70S6K Signaling	2.98E-02	
Oxidative Phosphorylation	3.27E-02	Phospholipidosis
Aryl Hydrocarbon Receptor Signaling	3.19E-02	
Bile Acid Biosynthesis	ns	
Mitochondrial Dysfunction	ns	Intersex, Phospholipidosis

Toxicology Functions	FDR	Associations
Liver Proliferation	2.11E-02	
Hepatocellular Carcinoma	2.11E-02	
Liver Cholestsasis	2.11E-02	Phospholipidosis
Liver Steatohepatitis	2.95E-02	Phospholipidosis
Liver Cirrhosis	ns	

Toxicology Lists	FDR	Associations
Oxidative Stress Response Mediated by Nrf2	2.95E-03	Phospholipidosis
Oxidative Stress	1.26E-02	Intersex
Xenobiotic metabolism	1.26E-02	Phospholipidosis, Vacuolar FCA
Mitochondrial Dysfunction	ns	Intersex, Phospholipidosis
Negative Acute Phase Response Proteins	ns	Intersex. Vacuolar FCA
Fatty Acid Metabolism	ns	Phospholipidosis

Significant Ingenuity pathways and toxicology functions and lists (FDR<0.05) among genes and metabolites significantly differentially expressed between sites (FDR<0.05) and between samples showing presence or absence of liver histologies.

### Molecular networks linked to fish morphometric indices represent the interface between metabolic and transcriptional networks

**Figure 1:**
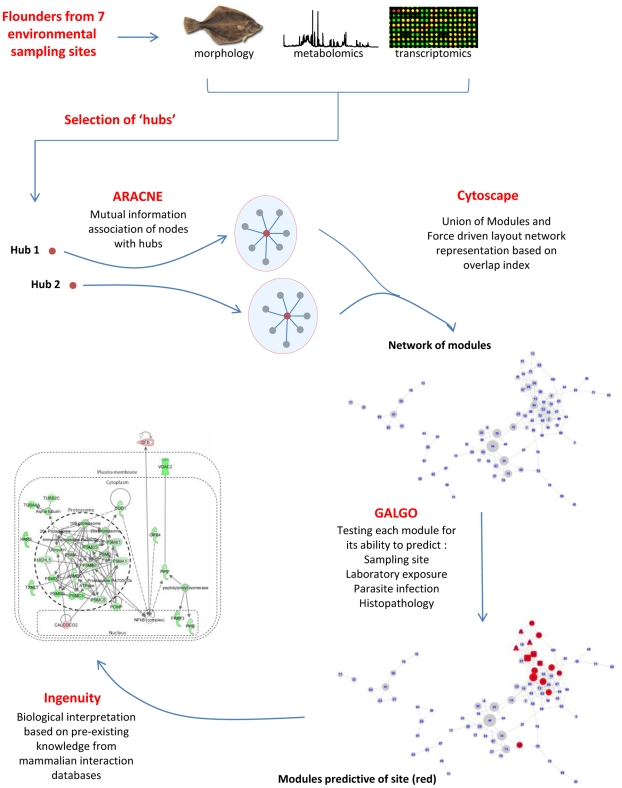
Experimental scheme. A schematic representation of the analysis workflow and the network inference methodology.

As there were clear relationships between geographical location, chemical exposure and molecular profiles of flounder livers, we proceeded to reconstruct a network model representing the relationships between transcriptomic and metabolomic data, morphological measurements, protein biomarkers and microsatellite markers (Figure 1). This network was constructed from all data, not limited to molecules that differed between sites.

**Figure 2:**
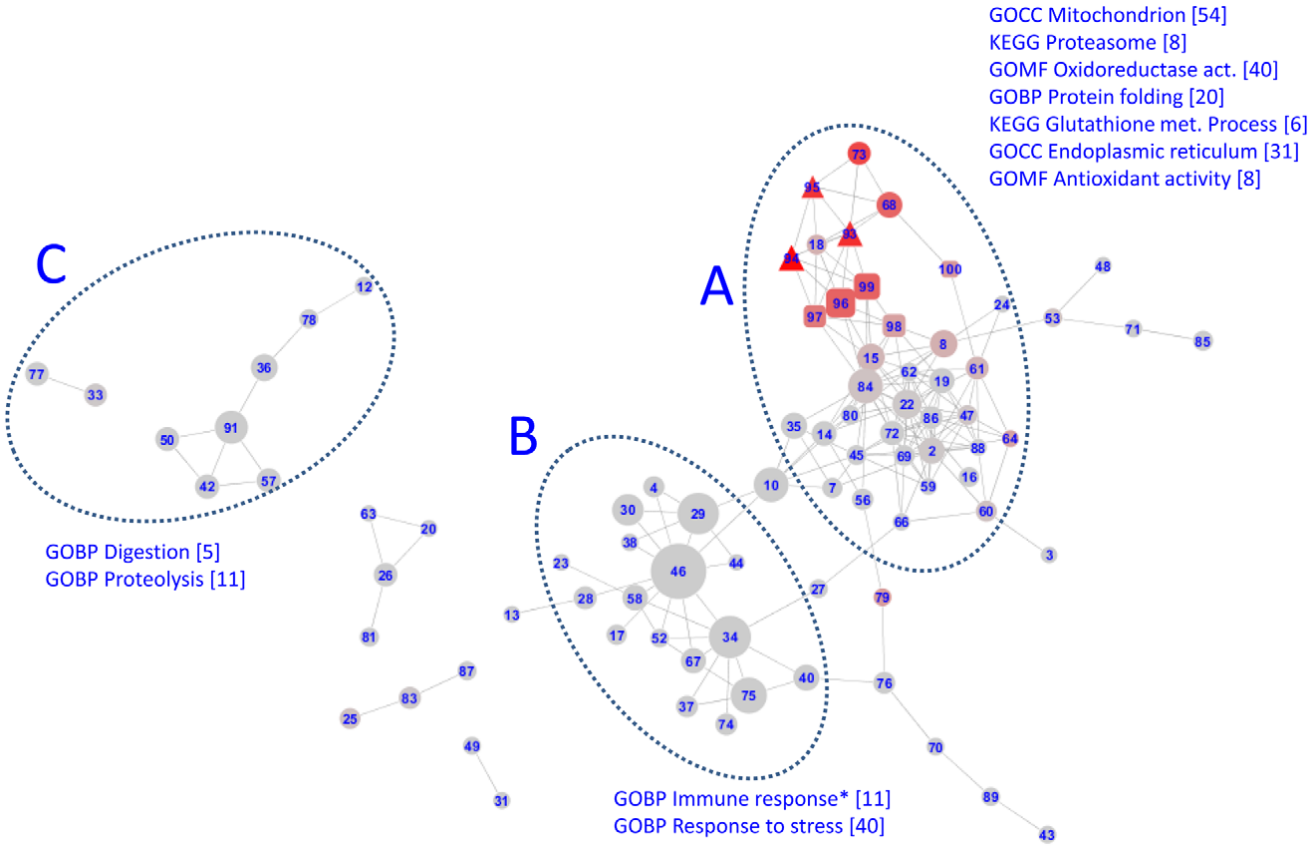
The network of modules that represents the flounder liver molecular state. Modules (consisting of transcripts, metabolite bins and morphological measurements) are numbered; sizes are proportional to the number of nodes within each module. Red colouring is proportional to the percentage of each module that consisted of metabolite bins. Modules whose seeds were transcripts are shown as circles; metabolites as triangles; morphological measurements as squares. Annotation terms significantly enriched (FDR<0.05 by DAVID) in areas of the network are shown (* = not statistically significant).

Inspection of the resulting network (Figure 2) showed that transcriptional and metabolic networks separated into two different areas of the network layout. Interestingly, modules whose hubs were fish morphometric measurements occurred exactly at the interface between these two areas and these modules contained metabolite (46%) and transcript (50%) measurements as well as fish morphometric measurements (4%).

Different areas of the inferred network (Figure 2) were characterised with different functional profiles. The modules close to the interface with metabolism (A) showed enrichment (FDR<0.05) for the annotation terms mitochondrion (GO:0005739), oxidoreductase activity (GO:0016491), endoplasmic reticulum (GO:0005783), protein folding (GO:0006457) and antioxidant activity (GO:0016209), and the two KEGG pathways hsa03050:proteasome and hsa00480:glutathione metabolism. The second sub-network (B) was enriched for immune response (GO:0006955) and response to stress (GO:0006950). The third sub-network (C) was enriched for proteolysis (GO:0006508) and digestion (GO:0007586).

### Identification of sub-networks predictive of geographical location and histopathological features

**Figure 3:**
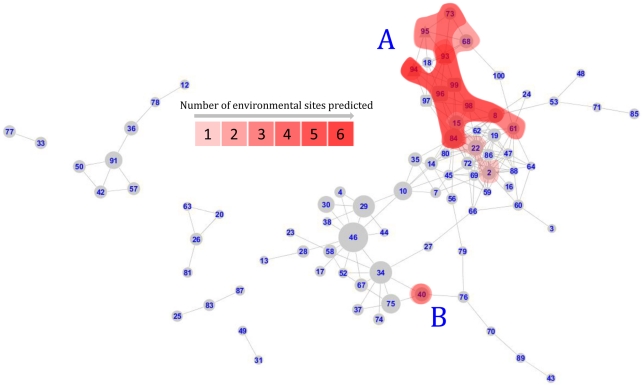
Modules predictive of environmental sampling sites. Modules in red predict membership of at least one environmental sampling site by GALGO with a sensitivity and specificity of >70%. The number of sites that each module could predict is illustrated by shading. Modules are split into a major group (A) and a minor group (B).

**Figure 4:**
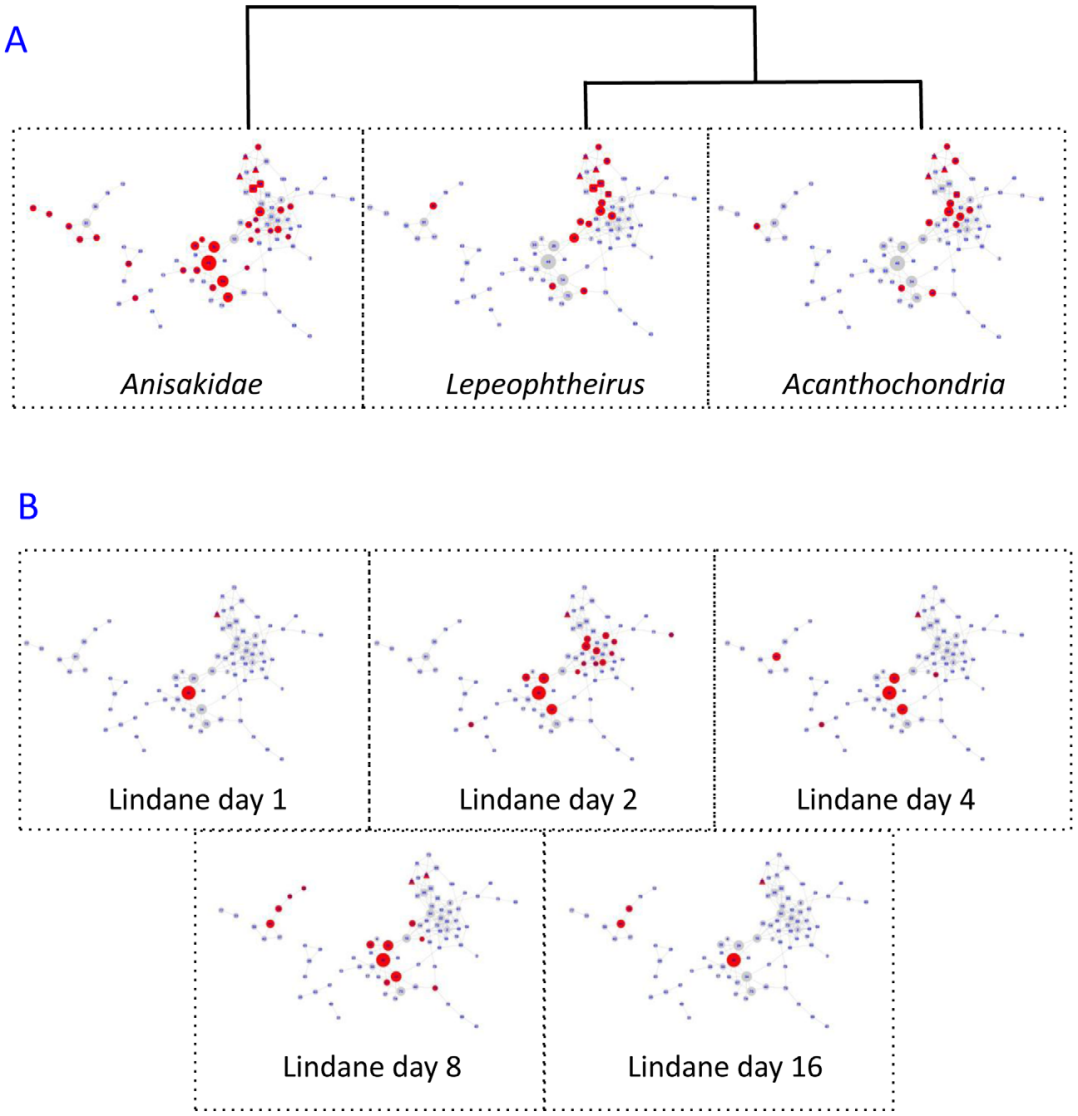
Modules overlapping with parasite infection and lindane treatment. A: Modules coloured red predict parasite infections using GALGO with a sensitivity and specificity of >70%. Clustering of profiles is shown. B: Modules coloured red significantly overlap (Fisher's Exact Test FDR<0.05) with transcripts significantly altering (ANOVA FDR<0.05, 2-fold change at each timepoint) in response to laboratory treatments of flounders with lindane over a 16-day time course.

Each individual network module was tested for its ability to predict geographic sampling sites (Figure 3), the presence of parasites (Figure 4A) and the presence of any of the liver histo-pathological abnormalities shown in Table 1H (Figure S2). Modules that were predictive of environmental sampling site were concentrated in two sub-networks. The larger (Figure 3, area A) was centred on the interface between metabolic and transcriptional networks and consequently included 14 modules consisting of morphometric indices as well as metabolites and transcripts (1% morphometric indices, 36% metabolite bins, 63% transcripts).

Modules that were predictive of parasitic copepod infection by *Acanthochondria* sp. and *Lepeophtheirus* sp. were similarly distributed in the network, but with additional modules localized in the sub-network B that were enriched in annotation to the immune response. Modules that were predictive of infection by *Anisakid* nematodes (Figure 4A) displayed a different profile, being more concentrated within group B. Hierarchical clustering of the profile of modules that were predictive of parasite infections showed that responses to copepod infections by *Lepeophtheirus* and *Acanthochondria* clustered together and were distinguished from responses to infection by the *Anisakid* nematodes.

### Laboratory exposures to individual chemicals mimic complex mixture responses in the environment

We have previously shown that there is a strong link between laboratory exposure to individual chemicals and flounder hepatic gene expression [8]. It was therefore reasonable to hypothesize that genes differentially expressed in laboratory exposures may map onto modules predictive of sampling location. Fisher's Exact Test was therefore used to identify modules where genes differentially regulated as a result of single chemical laboratory exposures were over-represented (these were determined by ANOVA FDR<0.05 over 16 day time-courses post-intraperitoneal injection). Responses to lindane are shown as an example in Figure 4B. This highlighted the temporal change in responses to toxicants, with the majority of overlapping modules occurring in both sub-networks A and B at early timepoints, followed by a shift towards sub-networks B and C at later timepoints. We have previously shown [8] that this temporal change is associated with an early induction of transcripts for chaperones, phase I and II metabolic enzymes, oxidative stress and protein synthesis that diminishes by the later timepoints and is replaced by induction of protein degradation, immune-function and inflammation-related transcripts.

The results for all treatments are illustrated graphically in Figure S2 E to L. All treatments showed overlap with modules in group A, at the metabolite/transcript interface, and this was clearest for cadmium, that only affected this area, apart from one module in group C. All other treatments showed overlap between responsive genes and group B modules to varying extents and all except estradiol and cadmium overlapped with at least two modules within group C. These results are supported by our previous study [9] in which we found that employing transcripts altered during laboratory exposures to a range of individual toxicants improved predictivity of environmental sampling sites.

### Environmental exposure to polluted sites recapitulates human liver pathophysiology

Having defined network modules predictive of geographical location, Ingenuity Pathway Analysis was used to elucidate the detailed structure of molecular pathways and their potential association with specific signatures of liver pathology. We performed these analyses under the hypothesis that the underlying response to chemical exposure would be consistent with what is known of human liver molecular pathophysiology. It was therefore expected that significant associations between the modules defined by our analysis and networks stored in the Ingenuity database would be informative of the underlying molecular mechanisms. We indeed observed a remarkable overlap between modules predictive of geographical location and modules containing genes whose transcriptional profile has been previously associated with liver fibrosis, cirrhosis and hepatocellular carcinoma in mammals.

**Figure 5:**
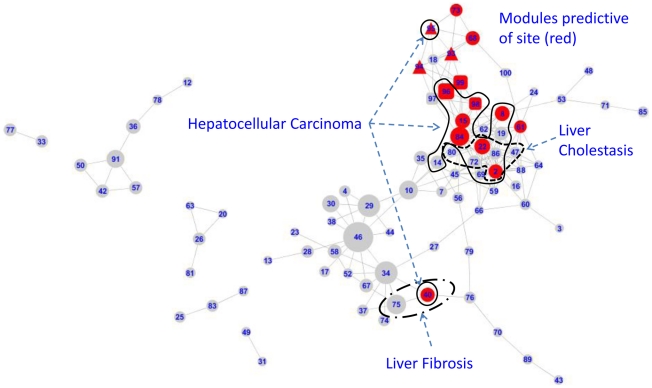
Modules associated with mammalian hepatotoxicity annotation. Modules predictive of sampling site are shown in red. Those associated with hepatocellular carcinoma are indicated by a solid line, those associated with liver cholestasis and liver fibrosis are indicated by dashed lines. Annotation terms were derived from Ingenuity.

Modules whose component genes related to hepatotoxicity are shown in Figure 5. The major group of site-predictive modules shows significant overlap with modules relating to liver cholestasis and hepatocellular carcinoma, whereas the secondary group overlaps with liver fibrosis. The annotation gained from Ingenuity, with key regulators inferred from networks based on interaction information, was combined and clustered in the TMEV software package using 5 different algorithms. These show (Table 4) that genes and metabolites a) involved in bile acid synthesis, transport and amino acid metabolism b) predictive of parasite infection c) linked to hepatocellular carcinoma, reproductive disorders and liver cirrhosis d) responding to oxidative stressors *tert*-butylhydroperoxide (*t*BHP) and cadmium, the hormone estradiol and rodent peroxisome proliferator perfluoro-octanoic acid (PFOA) are closely linked to differences between environmental sites. Additional relationships with inflammation, immune response, energy, fatty acids and nucleic acid metabolism, response to other toxicants and regulation by insulin, huntingtin, MYC and hepatocyte nuclear factor HNF4A were also highlighted. Functional analysis of the modules that were both site-predictive and associated with hepatocellular carcinoma showed significant overlap with mitochondrion, proteasome, tricarboxylic acid cycle, melanosome, protein dimerization activity, membrane-enclosed lumen, glutathione metabolism, coenzyme binding, microsome, translation, protein transport and carbohydrate catabolism (enrichment score >2, FDR<0.05).

**Table 4:** Annotation that clustered with environmental site prediction.

Annotation	Clustering
Bile Acid Biosynthesis	5
Molecular Transport	5
Cellular Compromise	5
*Lepeoptheirus* Infection	5
Amino Acid Metabolism	4
Cell Morphology	4
*Acanthochondria* Infection	4
Gly,Ser,Thr Metabolism	3
Nitrogen Metabolism	3
Reproductive Disorder	3
Hepatocellular Carcinoma	3
Liver Cirrhosis	3
*Anisakidae* Infection	3
Cd Anova	3
Cd d01	3
tBHP Anova	3
PFOA d04	3
E2 Anova	3
Inflammatory Disease	2
Infectious Disease	2
Nucleic Acid Metabolism	2
Citrate Cycle	2
Aroclor Anova	2
Huntingtin Regulation	2
Insulin Regulation	2
Myc Regulation	2
HNF4A Regulation	2
Lindane Anova	2
PFOA Anova	2
3MC Anova	2
Fatty Acid Metabolism	2

The number of algorithms clustering a given term with environmental site prediction is shown, out of a maximum of 5.

### Knowledge-based analysis of predictive modules reveals potentially novel response pathways

**Figure 6:**
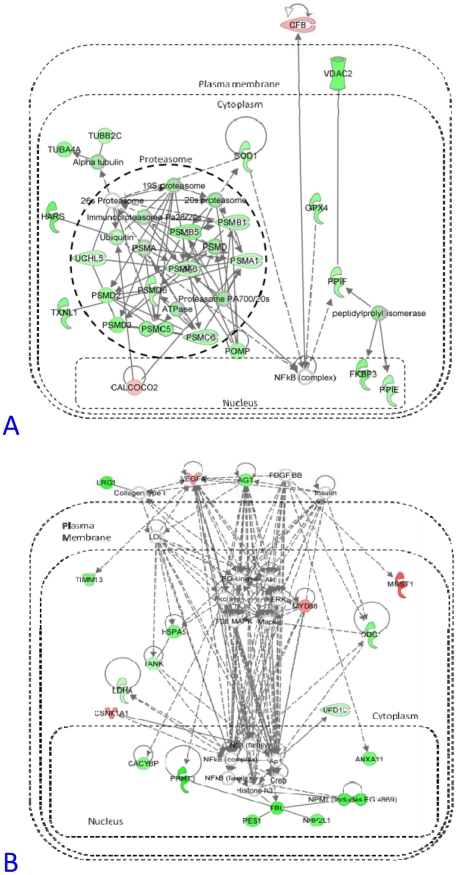
Networks derived from modules that were predictive of sampling sites. A– Most significant Ingenuity network derived from the union of modules that were highly predictive of sampling sites (5 or more), shown as major area A in Figure 3. B- Ingenuity network derived from the module that was predictive of sampling sites (3), shown as minor area B in Figure 3. Ingenuity networks are coloured by mean gene expression in Brunsbuttel fish versus Alde fish with red for induction more than 2-fold, dark green for repression more than 2-fold, pink or light green for changes less than 2-fold. Uncoloured nodes were predicted by Ingenuity.

The models we have developed are a high level representation of the molecular network's underlying response to environmental exposure. In order to generate specific hypotheses on the molecular pathways modulated during compensatory adaptation and toxicity further in-depth analyses of the specific interactions between genes and metabolites were performed. In this context, we combined the genes and metabolites represented in each group of predictive modules (Groups A and B in Figure 3) and input these to IPA software. The most statistically significant networks derived from each group are shown in Figure 6 and Figure S3, coloured by expression represented as a ratio between a highly polluted site and the reference site (Brunsbuttel versus Alde). The component genes and metabolites were clustered and the resulting expression profiles are shown in Figure S1. The Ingenuity networks are further described in Text S1 and are discussed below.

## Discussion

This is the first network level analysis of an environmental study integrating multilevel omic datasets. We discovered that the overall molecular state of the flounder liver (transcriptomics and metabolomics) is representative of the chemical contaminant burden of the sediments. Network reconstruction showed that the interface between transcriptional and metabolic network domains is linked to fish morphometric indices and is predictive of environmental exposure. In-depth analyses of predictive networks have identified putative novel pathways representative of responses to exposure. This approach provides a framework both for prediction of chemical pollutants in complex mixtures and for prediction of the health outcomes for exposed animals.

### The molecular state of flounder liver samples reflects chemical exposure

The chemical exposures predicted from CTD interactions were partly confirmed by chemical data (Table 2) despite the complexity of the environment, potentially including mixture effects, bioaccumulation and non-chemical stressors. Additional stressors were indicated that had not been chemically measured. Taking the Brunsbuttel site as an example, ethinyl-estradiol was a predicted contaminant and serum VTG protein, a canonical marker of endocrine disruption, was induced relative to the Alde (Table 1). Perfluorooctane sulfonic acid [16] and other persistent organic pollutants including PCBs, dieldrin and endosulfan [17] have been detected at elevated concentrations in the Elbe estuary and floodplain, and were all identified by our approach. Additional chemicals highlighted included systhane and vinclozolin fungicides, the halogenated aromatic hydrocarbon pesticide lindane, chlorine and tetradecanoylphorboyl acetate (TPA). It is uncertain whether these compounds are in fact present at this site as, for example, the presence of TPA appears unlikely. However TPA is a well-known tumour promoter [18], so detection of its associated gene expression changes might be viewed as a biomarker of effect, not necessarily of a specific exposure. At a number of sites flavonoids and flavonols, such as epicatechin gallate, were predicted, potentially indicating plant-derived exposures not of anthropogenic origin. At Morecambe Bay and the Tyne the prediction of paraquat perhaps reflected an oxidative stress response rather than the presence of this particular compound.

These results support the use of a knowledge-based approach to infer chemical exposure profiles from molecular responses and validate the underlying assumptions in the study. Predictions from interrogation of the CTD database (Table 2; Table S3) differed between sites suggesting that the approach can be sufficiently sensitive to specific differences in the exposure profiles. However, we do not propose that these associations necessarily indicate the presence of each specific contaminant at each site, for example ‘tobacco smoke pollution’ in the Mersey, we instead hypothesise that these represent the effects of related stressors, for example, AhR inducers at the Mersey site.

### Interpretation of the molecular interaction network

The development of a modular network, representing the integration between molecular and physiological readouts, provided us with an interpretive framework to analyse the complex molecular signatures linked to exposure. One of the most interesting findings is that the modules that predict environmental exposure with greatest accuracy represent the interface between metabolite and transcriptional networks and link to higher level indicators of fish health, such as condition factor and hepatosomatic index (Figure 2).

Consistent with this observation, network modules at the interface between metabolite and transcriptional networks were also differentially regulated in response to single chemical laboratory exposures. It should be borne in mind that the environmentally sampled fish have been chronically exposed to pollutants, and that chronic exposure can result in different responses than acute exposure [19], [20]. In addition bioavailability, mixture effects, metabolism and bioaccumulation affect compound-specific responses within the livers of these fish. This is illustrated by the modules containing genes that responded to 16-day treatments of flounder with individual toxicants (Figure 4 B, Figure S2). While all toxicants induced changes in the metabolic-interface genes, they also affected the secondary area of the network that related more to acute stress and immune response (Figure 2, area B), in contrast to the differences between environmental sites, where only one module (40) in this area was affected.

The characterisation of transcripts and metabolites that differed between sites was undertaken to provide insights into the molecular mechanisms that they describe, and to inform on the potential health outcomes for the fish. Canonical pathways that contributed to these differences included those relevant to metabolism of toxicants; AhR signalling, metabolism of xenobiotics by cytochromes P450, the NRF2-mediated oxidative stress response, glutathione metabolism and bile acid bioysnthesis (Table 3). Together these describe phase I and II metabolism of xenobiotics, such as aromatic hydrocarbons, and their excretion via the bile. Additional endobiotic metabolic pathways were affected. Changes in glycolysis, pyruvate metabolism, the citric acid cycle and oxidative phosphorylation implied disturbances to the energy pathways of the liver that could reflect the energetic requirements of xenobiotic metabolism and lead to further metabolic disruption. Changes in amino acid synthesis and proteasomal protein degradation also indicated reorganisation of metabolism.

This change in metabolic state and gene expression could be viewed as a successful compensatory response to toxicants and thus of little concern for the health of individual fish and these fish populations. Further examination of the annotation of transcripts and metabolites differing between sites implied that this hypothesis was false. As illustrated in Figure 5, and shown in Tables 3 and 4, there is a remarkable overlap between site-predictive modules and modules associated with hepatocellular carcinoma (HCC). Additionally, liver cholestasis -annotated modules overlapped with HCC and site predictive modules and this area of the network was highly associated with bile acid biosynthesis. Apart from this metabolic interface group only one other module (module 40) was predictive of site. This was also associated with hepatocellular carcinoma, and additionally with liver fibrosis, indicative of chronic liver damage, and occurred in an area of the network associated with inflammation. Therefore flounders inhabiting differentially contaminated sites show transcript and metabolite changes that have been associated with liver carcinogenesis in mammals. A question remains as to whether this simply represents the detection of HCC in the liver samples, as histopathology data were unavailable for the fish sampled off Germany. By comparison with studies of tumours from the closely related flatfish dab (*Limanda limanda*) this does not appear to be the case. In dab tumours the metabolites choline, phospocholine and glycine were reduced in concentration and lactate increased, an indication of the switch to anaerobic metabolism in the bulk tumours [21]. In, for example, the Brunsbuttel samples compared with non-tumour bearing Alde fish, choline, phosphocholine and glycine increased, and lactate decreased. Additionally, transcripts for ribosomal proteins showed co-ordinated induction in bulk tumours from dab, indicative of proliferation [22], but no such induction was apparent from the present samples. The changes in gene expression and metabolites detected in this study do not recapitulate those found in bulk tumours, and may be viewed as indicating either an earlier stage of tumourigenesis or a permissive micro-environment in which hyperplastic tissue may form and lead to tumour formation.

### Network analysis reveals potentially novel response pathways

Ingenuity networks, based on mammalian interaction data, permitted more detailed biological characterisation of the site-associated modules. Complete pathways were not recapitulated by these analyses, as only a minority of the transcripts and metabolites from flounder liver were examined. Nevertheless, the analyses highlighted important processes and inferred key regulators. Here the most significant network derived from site-predictive modules is discussed in detail and additional networks are discussed in terms of their key inferred regulators.

**Figure 7:**
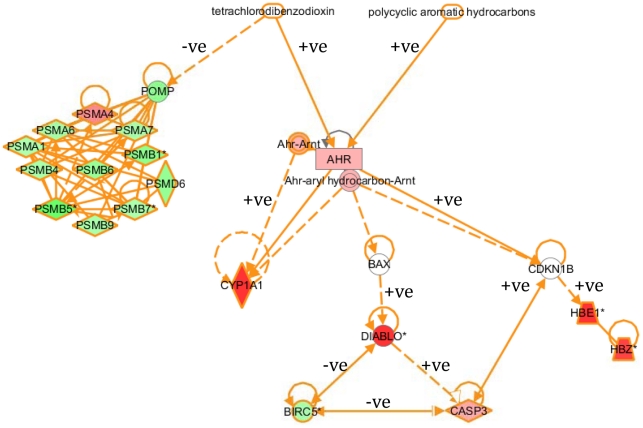
Relationship between the AhR pathway and proteasome. Relationship between the AhR pathway and proteasome, generated within Ingenuity, coloured deep red or green for changes exceeding 2-fold up and down respectively between Brunsbuttel and Alde fish, and light red and green for those less than 2-fold.

The most striking finding from the Ingenuity analyses was the co-ordinated repression of proteasomal subunit genes at the Brunsbuttel site (Figure 6A; Figure S3A1). This was not so marked at other sites (Figure S1), indeed at Morecambe Bay these genes were induced in comparison with the Alde fish. Proteasome maturation protein (POMP) has been found to be a critical regulator of proteasomal activity [23] and has been shown to be repressed by the halogenated aromatic hydrocarbon 2,3,7,8-tetrachlorodibenzodioxin (TCDD) in an AhR-independent manner [24]. Although TCDD concentration was not measured, the mean expression of proteasomal genes was inversely correlated (r = −0.79) with fish liver PCB concentrations but did not correlate well with sediment PAH or PCB concentrations. Tyne fish, for example, displayed relatively high proteasomal gene expression and had low liver PCB but high PAH concentrations (Table 1, Figure S1). Therefore the repression of proteasomal genes may represent a halogenated aromatic hydrocarbon-related response (Figure 7). In trout *Oncorhynchus mykiss*, a proteasome inhibitor reduced PAH-dependent CYP1A induction [25], in contrast to mammalian studies [26]. This difference may contribute to the lower inducibility of CYP1A in flounder in comparison with many mammals. Ingenuity analysis also predicted an interaction between the proteasome and NF kappa B, a key regulator of mammalian hepatocarcinogensis [27]. The proteasome represses NK kappa B activation, and potentially disruption of proteasomal activity could have extensive additional effects on intracellular protein levels due to its role in the degradation of numerous proteins. We found no significant changes in NF kappa B gene expression between sites, and the consequences of putative activation at the Brunsbuttel site, and repression at the Morecambe site, due to changes in the proteasome, are difficult to predict, as in the early stages of carcinogenesis NF kappa B can have a protective effect, whereas in later stages it can promote tumourigenesis [27].

From the Ingenuity networks a number of key regulatory molecules were inferred. These included insulin (Figure 6B, Figure S3A2, S3A4, S3B1), estrone, luteinizing hormone (LH) and follicle stimulating hormone (FSH) (Figure S3A3 and S3A6), platelet derived growth factor beta (PDGFBB) (Figure 6B, Figure S3A2, S3B1), transforming growth factor beta (TGF-beta) (Figure S3A8), vascular endothelial growth factor (VEGF) (Figure 6B, Figure S3A7, S3B1), tumour necrosis factor (TNF) (Figure S3A5), and angiotensinogen (Figure 6B, Figure S3B1).

Insulin, in fish as in mammals, is a key hormonal regulator of energy, glucose and lipid metabolism, all pathways that were identified as affected by sampling site. By the Ingenuity networks it was linked to protein kinases, metabolites (including glucose and lactate) and the glucose transporter SLC2A4. The most obvious explanation for changes in insulin and related parameters would be differences in diet between fish from different sites. Amino-acid levels are more important regulators of insulin in carnivorous fish such as the flounder than sugars [28]. Dietary parameters would be expected to be highly variable depending upon recent feeding history of the fish, which was unknown for these individuals. However, insulin can also be modulated by exposure to toxicants including organophosphates [29] that was suggested to lead to an increase in lipogenesis, in agreement with our observations of phospholipidosis in fish from polluted sites (Table 1). Mild estrogenic endocrine disruption was suggested by VTG induction in Brunsbuttel fish, and networks shown in Figures S3A6 and S3A3 inferred that estrogen receptor alpha (ESR1), FSH and LH target genes were modulators of the different responses between sampling sites. ESR1 and HNF alpha were linked in Figure S3A6 and both are involved in hepatic cholestasis, indeed EE2-induced hepatotoxicity has been linked to alterations in bile acid biosynthesis in mice [30].

PDGFBB is the dimeric form of platelet derived growth factor beta (PDGF-B). Notably, PDGF-B over-expressing mice spontaneously developed liver fibrosis [31], and PDGF-BB was inferred as part of the network deriving from the liver fibrosis-annotated module 40 in our analysis. Additionally PDGF-B over-expressing mice developed hepatocellular carcinoma in response to phenobarbital and diethylnitrosamine treatment and induced TGF-beta and VEGF expression. TGF-beta was inferred to be an important regulator in site-specific responses (Figure S3A8) and is a well-known mediator of cancer initiation, progression and metastasis, via interaction with the inflammatory response [32]. Furthermore, the pro-inflammatory cytokine TNF-alpha, an initiating signal for the innate immune response in fish as well as mammals [33], was also identified by Ingenuity analysis (Figure S3A5). Release of TNF alpha from Kupffer cells leads to hepatocyte cell death, regeneration and fibrosis that can lead to hepatocellular carcinoma [34]. VEGF, best known as a stimulator of angiogenesis, was also highlighted in both the fibrosis-related and carcinoma-related sections of the network, and was linked with cell cycle, oncogenes and tumour suppressor genes (CDKN1A, TP53, MYC). Angiogenesis is a key requirement for the transition from fibrosis to hepatocellular carcinoma [35].

Angiotensinogen (AGT) is the precursor of angiotensin and was found to be repressed at all sites in comparison to the Alde reference site (Table S1). Angiotensin is a signal for vasoconstriction in mammals and in fish its expression is related to osmoregulation [7] with repression in liver in response to higher salinity. As the sampling sites differed in salinity, alteration of AGT transcription was not a surprising finding. As shown in Figure 6B, AGT was a member of the fibrosis-related module 40 and was predicted to form part of a complex network with VEGF, PDGF and intracellular kinases. Angiotensin has indeed been linked to stimulation of inflammatory liver fibrosis [36], via fibroblast proliferation and production of inflammatory cytokines and growth factors, including TGF-beta. Inhibition of the angiotensin system by antagonism of its receptor [37] or inhibition of angiotensin-converting enzyme [38] has been shown to reduce hepatic fibrosis.

VEGF, TGF beta, TNF alpha, PDGF and AGT are all intimately related to the progression of fibrosis to cirrhosis and hepatocellular carcinoma in mammals. These molecules were all highlighted as important regulators of the differences between molecular profiles of flounder livers from different sampling sites using an unbiased approach combining network inference and predictive algorithms.

A combination of omics, multiple biomarkers and bioinformatics were used to identify and characterise hepatic molecular changes between fish sampled from several environmental sites. Based on these data, parasite infection, fish morphology and genetics do contribute to the differences between sites, but do not explain the majority of changes seen. For example, within-site tests showed that morphometric parameters and parasite infections could be significantly associated only with a small proportion (<3%) of the gene expression differences between sites (Table S1, Table S4). Taken as a whole with our previous studies [8], [9], we find that anthropogenic chemical contamination of the marine environment is a major factor in explaining the molecular differences between fish sampled from these sites.

The different methodologies employed displayed different strengths and weaknesses. Histopathology was a good guide to broad levels of pollution effect, but provided little information upon the nature of the contaminant profile. Protein biomarkers and enzyme activities were useful for categorising sites by major classes of toxicant, but gave little information on the potential health outcomes. ^1^H NMR metabolomics showed low technical variability, and metabolite profiles alone were more predictive of sampling site than gene expression profiles alone, however the annotation of metabolites is not yet well advanced, limiting the functional information currently available. Transcriptomics exhibited higher variability than metabolomics, but was more informative due to better annotation. Overall the methodologies were highly complementary, allowing analyses that would be impossible if one were limited to a single technique.

The gene expression signatures associated with fish from each sampling site were used to predict the presence of chemical contaminants using the CTD gene expression-chemical interaction database. Mixture effects, other environmental influences and the similarity of certain stressors, such as the metals, might be expected to confound this approach. Additionally the incomplete nature of the flounder microarray and the CTD database and the limited numbers of samples for certain sites, which is a common issue in field studies, reduce the potential of this analysis. Therefore we did not expect to predict all environmental contaminants by this method. While this approach was useful with the current dataset, it may be expected to improve in future as both the CTD database and transcriptomic data become more comprehensive.

Data integration and network analyses were essential; both to predicting health outcomes and to identifying and examining affected biological pathways. They allowed visualisation of the highly complex dataset and facilitated comparison of the effects of different stimuli upon the model system. Modules associated with specific parameters could then be examined in detail, utilising interaction databases (Ingenuity) for further characterisation. Detailed examination of these networks illustrated the changes detected by broader classification of modules by annotation terms. In addition to potential interactions with diet and salinity, the majority of networks contained key regulators of inflammation, hepatic fibrosis and hepatocellular carcinoma. Therefore we propose that network biology approaches can lead to the identification of health impacts of environmental pollutants upon non-model organisms.

The molecular differences between reference and contaminated sampling sites were associated with carcinogenesis, and this outcome is supported by previous histopathology [10], [39]. Flatfish hepatic histopathology has long been associated with chemical contamination [39] and our results demonstrate the linkages between toxicants and histopathology via alterations in molecular signalling pathways and metabolism.

## Methods

### Fish sampling

The sampling sites employed in this study were: In UK waters; on the Irish Sea, the Mersey estuary, at Eastham Sands, Liverpool (lat 53°19N, long 2°55W) and Morecambe Bay (lat 54°10N, long 2°58W); on the North Sea, the Alde estuary, Suffolk (lat 52°95N, long 01°33E) and the Tyne estuary at Howdon, Tyne and Wear (lat 54°57N, long 1°38W): In North Sea waters off Schleswig-Holstein, Germany; the Elbe estuary at Cuxhaven (lat 53°53N, long 08°15–19E) and Brunsbuttel (lat53°52N, long 09°09–10E) and off Helgoland (lat 54°06N, long07°15–08°00E). Adult European flounders (*Platichthys flesus*) were caught during statutory monitoring programs carried out by the Centre for Environment, Fisheries and Aquaculture Science (Cefas) at UK sites in April 2006 and by AWI Bremerhaven at FRG sites in October 2004 and April 2005. Fish were caught using beam trawls and held in tanks of flowing sea water onboard ship and were dissected either onboard ship or on return to shore. Livers were immediately removed and 100 mg samples for microarrays and metabolomics and 200 mg samples for biomarker assays were flash frozen in liquid nitrogen, with liver slices taken for histopathology. Blood was extracted and stored at 4°C overnight before plasma preparation for vitellogenin (VTG) analysis. Fin clips (1 cm^2^) were preserved in 70% ethanol at 4°C for genotyping. After sexing, livers from males that were 12 to 34 cm long were used for further analyses, this sample set included n = 20 for Alde, n = 16 for Tyne, n = 22 for Mersey, n = 23 for Morecambe Bay, n = 22 for Helgoland, n = 24 for Cuxhaven and n = 48 for Brunsbuttel. Fish lengths and weight, condition factor (K; body wt/length^3^×100), liver weight and hepatosomatic index (HSI; liver wt/body wt×100) were determined for all samples, gonad weight and gonadosomatic index (GSI; gonad wt/body wt×100) for FRG fish only.

### Chemistry

Chemical determinations were carried out on sediment samples and independent sets of flounder liver samples from the same samplings by Cefas and Deutsches Ozeanographiches Datenzentrum, Germany and submitted to the International Council for the Exploration of the Sea (ICES), Copenhagen, Denmark as part of the national marine monitoring programmes. UK data was analysed from that collected as part of the Clean Safe Seas Environmental Monitoring Programme (CSEMP) and archived in the UK's Marine Environment Monitoring and Assessment National database (MERMAN). For sediment; metal concentrations (Al, As, Cd, Cr, Cu, Fe, Hg, Li, Mn, Ni, Pb and Zn); polycyclic aromatic hydrocarbons (PAHs) (anthracene, benzo[a]anthracene, benzo[a]pyrene, benzo[ghi]perylene, chrysene/triphenylene, fluoroanthrene, indo[123-c]pyrene, naphthalene, phenanthrene and pyrene); total organic carbon and polychlorinated biphenyls (PCBs) (congeners CB28, 52, 101, 118, 138, 153 and 180) were determined and the sum of PAHs and the sum of ICES 7 priority PCBs calculated for all sites. For flounder livers; metals (As, Cd, Cr, Cu, Fe, Hg, Ni, Pb, Se and Zn) and PAHs (acenaphthylene, acenaphthene, benzo[a]anthracene, C1-, C2- and C3- naphthalene, C1-phenanthrene/anthracene, chrysene, fluoroanthrene, fluorene, naphthalene, phenanthrene and sum of PAHs) were determined for Alde, Tyne and Mersey fish, with partial metal concentration data for Morecambe Bay, Helgoland, Cuxhaven and Brunsbuttel samples. Polychlorinated biphenyls (PCBs) (congeners CB28, 52,101, 118, 138, 153, 180 and sum of ICES 7 PCBs) were determined for liver samples from all sites. Data are available from the Merman database (http://www.bodc.ac.uk/projects/uk/merman/).

### Histopathology

UK flounders were examined for external lesions, liver gross appearance and parasite infection. Liver pathology was assessed according to the criteria of Feist *et al.* [40]. Sections of liver tissue were removed, placed into individual histological cassettes, transferred to 10% neutral buffered formalin and processed for histopathology as described previously [11]. The presence of toxicopathic lesions, foci of cellular alteration, benign neoplasia, malignant neoplasia and non-specific inflammatory lesions was determined.

### Biomarker assays

Plasma vitellogenin (VTG) concentrations (mg/ml) were determined by the method described by Kirby *et al* [41]. Hepatic metallothionein (MT) concentration (µg per mg) and glutathione reductase (GR) (nmol/mg), glutathione-*S*-transferase (GST) (µmol/mg) and ethoxyresorufin-*o*-deethylase (EROD) (pmol/mg) activities were determined by the methods of George and Young [42]. These assays were carried out for all except Cuxhaven and Helgoland fish.

### Genetics

Flounder fin-clip samples (n = 50) from all sites were surveyed for six neutral microsatellite markers (all polymorphic) and 13 detoxification gene-associated size variants within introns of flounder cytochrome P450 1A (CYP1A) [43], GST-A [44] and peroxisome proliferator activated receptors (PPAR) alpha, beta and gamma [45]. Following targeted PCR spanning each polymorphic site; DNA fragments were detected and sized by fluorescent capillary electrophoresis (Beckman CEQ8800 sequencer). Chromatogram files were individually inspected, and alleles were identified/scored manually. Four standard flounder DNA samples were analysed in each genotyping run (96 sample plate) to maintain scoring consistency. Standard genetic analyses for both single and multi-locus conformance to Hardy-Weinberg expectations within samples and examination of potential allelic differentiation among sites were undertaken using GENEPOP [46]. PHYLIPv3.5 software [47] was then employed to compute and compare four different measures of genetic distance (Nei's standard and Da distances; Cavalli-Sforza chord distance; Reynolds distance) and to construct unrooted neighbour-joined dendrograms (branch points being bootstrap-supported).

### Transcriptomics

The GENIPOL flounder cDNA microarray has been described previously [48], [49]. The methods and design were similar to those employed in earlier experiments, with minor modifications [8], [9]. Briefly, liver tissue from individual flounders was homogenised in a methanol/water mixture [50] and aliquots were taken for both metabolomics and transcriptomics. Liver homogenates were used to prepare total RNA (Qiagen, Crawley, UK), reverse-transcribed to cDNA and labeled with Cy5-dCTP fluorophore (GE Healthcare, Amersham, UK). Labeled cDNAs were individually statically hybridised overnight to the microarray versus a common Cy3-labeled synthetic reference, before stringent washing and scanning (Axon 4000B; Molecular Devices, Wokingham, UK). Data were captured using Genepix software (Molecular Devices), and each slide was checked in detail, with spots showing poor morphology or arrays showing gross experimental artefacts being discarded. The data consisted of local background-subtracted median 635 nm intensities. MIAME-compliant gene expression data are available from ArrayExpress under accession E-MTAB-396. As the microarray is redundant, CAP3 clustering [51] had been used to identify contiguous sequences [48].

### Metabolomics

For metabolomics, liver homogenate aliquots were further extracted individually using methanol/chloroform/water (2∶2∶1.8 final volumes) [50], [52]. One-dimensional ^1^H NMR spectroscopy was performed upon the hydrophilic fraction as previously described [53]. Briefly, NMR spectra were measured at 500.11 MHz using an Avance DRX-500 spectrometer and cryogenic probe (Bruker, Coventry, UK), with 200 transients collected into 32k data points. NMR data sets were zero-filled to 64k points, exponential line-broadenings of 0.5 Hz were applied before Fourier transformation, and spectra were phase and baseline corrected, then calibrated (TMSP, 0.0 ppm) using TopSpin software (version 1.3; Bruker). The subsequent processing and statistical analyses of the NMR data have been described in detail in a previous study [53]. Briefly, taurocholic acid, an abundant bile acid with highly variable concentration in the liver extracts was subtracted from each spectrum using Chenomx NMR metabolomics software (version 4.6; Chenomx, Edmonton, Canada). Next, residual water was removed, each spectrum was segmented into 0.005 ppm bins, and the total area of each binned spectrum was normalized to unity so as to facilitate comparison between the samples. Subsequently to statistical analyses, significantly changing metabolite ‘bins’ were identified as particular metabolites by comparison with spectral libraries of reference compounds and were annotated with PubChem CID accessions (NCBI).

### Data analyses

Microarray data were filtered to remove spots where 20% or more of the data were undetectable over all samples and background-subtracted intensity values of 0 or below were set to 0.5. Data were log2 transformed, quantile normalised and de-noised by a) removing data where SD/mean was more than 0.9 and b) removing data where maximum–minimum was less than 1.5. Missing data were estimated using MetaGeneAlyse probabilistic principal components analysis (PCA) algorithm [54]. Array slide batch effects were resolved using an empirical Bayes correction [55]. A representative clone with greatest average expression across all samples was chosen for each contiguous sequence cluster where the Pearson correlation score was greater than 0.6 to other members of the cluster. Where the correlation failed to pass this cut-off, data were discarded. The noise level for each metabolomics NMR spectrum was estimated by dividing the spectrum into 32 regions and calculating the smallest bin SD for each region and multiplying this by 3. These results were used to de-noise the data [56]. Data from metabolomics, transcriptomics and fish measurements (K, length, weight, liver weight, HSI) were then combined where all were available. The final data set therefore consisted of n = 15 for Alde, n = 9 for Tyne, n = 9 for Mersey, n = 13 for Morecambe Bay, n = 21 for Helgoland, n = 23 for Cuxhaven and n = 36 for Brunsbuttel. An additional dataset was generated for the omics samples that also possessed genetic data.

Normalised combined microarray and metabolomic data were input to Genespring GX 7.3.1 (Agilent Technologies, Santa Clara, CA, USA). Statistically significantly changing genes were found by 1-way ANOVA with a multiple testing correction [57] for a false discovery rate (FDR)<0.05, and with Welch T-tests employing the same FDR. Fold change cutoffs of 1.5-fold were additionally applied. A classification algorithm was used to compare previous data [9] with the current data; this employed the Support Vector Machines algorithm within Genespring with the Kernel Function Polynomial Dot Product (Order 3), a Diagonal Scaling Factor of 0, for all genes passing QC cut-offs in both experiments. Gene ontology (GO) analyses were carried out within Blast2GO [58], [59] employing the GOSSIP package [60]. As flounder is a non-model species, genes were annotated with gene symbols of their putative human orthologs, found by employing a Conditional Stepped Reciprocal Best Hit approach between flounder and zebrafish (*Danio rerio*) and human transcriptome databases, similar to Herbert *et al.* [61], with additional manual curation.

Chemical-gene expression interactions were downloaded from the Comparative Toxicology Database (CTD) [15], for all annotated genes. These represent a database of the previous literature on chemical-gene expression interactions. The chemical-gene pairs from this list were segregated into inducers and repressors, duplicates were removed, and the two lists uploaded into TMEV [62], thereby annotating each gene with its ‘chemical inducers’ and ‘chemical repressors’. Lists of genes (ANOVA, FDR<0.05, fold change versus Alde>1.5; illustrated in Table S1) were interrogated for enrichment of chemical associations using EASE (Expression Analysis Systematic Explorer) within TMEV, and FDR calculated. Where associations were found between an inducing chemical and induced genes and also between the same chemical acting as a repressor and repressed genes, FDRs were multiplied to produce a final FDR value. Lists of genes and metabolites were additionally interrogated by Ingenuity Pathway Analysis (Ingenuity IPA 8.5; Ingenuity Systems, Redwood City, CA, USA), employing Human Gene Organisation (HUGO) gene identifiers and PubChem CID compound identifiers, with statistical tests using Benjamini and Hochberg multiple testing corrections.

The overall approach taken for network construction and analysis is shown in Figure 1. It is conceptually sub-divided into: 1) Selection of network hubs: 2) Construction of a fully connected network: 3) Identification of network modules representing the neighbourhood of the hubs: 4) Assembly of the final modules and graphical representation: 5) In-depth analysis of gene interactions using Ingenuity Pathway Analysis (IPA) software.

The network was constructed from all measured variables, including transcript, metabolite, morphometric, protein biomarker and genetic data. Within the network each individual variable is described as a node. We selected 99 ‘hub’ nodes representing transcripts with known toxicological and regulatory relevance in order to identify the molecular network representing the interactions between these hubs and all the other nodes in the multi-level dataset. In addition, morphometric indices and metabolite peaks were also included in the list of hubs to represent the complexity of the metabolic networks, which, we reasoned are likely to closely influence liver physiology. The network inference methodology ARACNE [13] was used to create the network. Statistically significant interactions were selected on the basis of mutual information between the nodes at cut-off of P<1e-6. We defined 99 modules derived from each selected hub and its neighbourhood. Many nodes were present in multiple modules. The overlap index was calculated between each pair of modules by dividing the number of overlapping nodes by the total number of nodes in the smaller module. The final network was then constructed as the union of all network modules and visualized using a force driven layout available in the software application Cytoscape [63]. In the final network, the edge distances between the modules are relative to the overlap index and the node sizes are relative to the size of the module. We also compared this strategy to develop mutual information-based modules from hub variables with a more complex method [64], shown in Text S1, and discovered that they both gave similar results.

Subsequently the multivariate selection algorithm GALGO [65] was applied to each module to determine its predictivity for parameters including fish sampling site, parasite infection, and the presence or absence of liver pathologies. The cut-off employed for identification of predictive modules was >70% specificity and >70% sensitivity. Genes were annotated with HUGO identifiers for their putative human orthologs. DAVID v 2008 and v6.7 [66], [67] was used to classify module genes and groups of modules inferred from the topology of the module graph, by their shared Gene Ontology (GO) and other annotation terms. Flounder laboratory treatment data was employed to relate gene expression changes seen in the environmental samples to those elicited by model toxicant treatments. These treatments consisted of a single intraperitoneal injection with cadmium chloride (Cd, 50 µg/kg), 3-methylcholanthrene (3-MC, 25 mg/kg), aroclor 1254 (50 mg/kg), *tert*-butyl-hydroperoxide (*t*BHP, 5 mg/kg), lindane (25 mg/kg), perfluoro-octanoic acid (PFOA, 100 mg/kg), estradiol (l0 mg/kg) and furunculosis vaccine (killed *Aeromonas salmonicia*, Aquavac Furovac 5; 1 ml/kg) with gene expression monitored over a 16-day timecourse versus appropriate controls. Full details are shown in Williams *et al.* [48], Williams *et al.* [8] and Diab *et al.* [49], with data available from ArrayExpress under accessions E-MAXD-32 and E-MAXD-38. Overlap between module genes and genes differentially expressed by toxicant treatments over the 16 day timecourse, ANOVA, FDR<0.05 was determined by Fisher's Exact Test with a cut-off of P<0.01. Modules and groups of modules were interrogated by Ingenuity Pathway Analysis. Key regulatory molecules were inferred from networks generated within Ingenuity, that also output functional enrichment within the lists of nodes (P<0.05). Modules were annotated with associated diseases, functions, canonical pathways, toxicity and hepatotoxicity terms within Ingenuity and with inferred key regulators, as well as with environmental and parasitological predictivity and overlap with laboratory treatment data to produce a binary matrix. This was clustered within TMEV [62] using hierarchical clustering, SOTA self organising tree, K-means, QT and SOM self organising map algorithms. Grouped modules that were predictive of sampling site were subjected to Ingenuity analyses and were overlaid with Brunsbuttel expression data relative to Alde, the reference site. These genes and metabolites were subjected to K-means clustering within TMEV and the clusters functionally annotated using DAVID.

## Supporting Information

Figure S1 **Expression of site-predictive genes and metabolites.** Expression profiles of the site-predictive genes and metabolites shown in Figure 6, separated by K-means clustering and functionally annotated within DAVID.
(DOCX)

Figure S2 **Modules overlapping with histopathology and chemical treatments.** A to D – modules coloured red predict parasite infections and presence of histopathological liver abnormalities using GALGO with a sensitivity and specificity of >70%. E to L - modules coloured red significantly overlap (Fisher's Exact Test FDR<0.05) with transcripts significantly altering (ANOVA FDR<0.05) in response to laboratory exposures of flounders to individual stimuli over 16-day time courses.
(PPTX)

Figure S3 **Networks derived from modules that were predictive of sampling sites.** A1 to A8 – Ingenuity networks derived from the union of modules that were highly predictive of sampling sites (5 or more), shown as major area A in Figure 3. B1 Ingenuity network derived from the module that was predictive of sampling sites (3), shown as minor area B in Figure 3. Ingenuity networks are coloured by mean gene expression in Brunsbuttel fish versus Alde fish with red for induction more than 2-fold, dark green for repression more than 2-fold, pink or light green for changes less than 2-fold. Uncoloured nodes were predicted by Ingenuity.
(PPTX)

Table S1 **Gene expression and metabolic changes.** Identifiable transcripts and metabolites that were significantly different between sampling sites (ANOVA FDR<0.05). Best viewed in MS-Excel.
(XLSX)

Table S2 **Correlations of omic data with other variables.** Correlations between gene and metabolite expression and chemistry data; comparisons of gene and metabolite expression with presence or absence of liver pathologies; comparisons of gene and metabolite expression with metallothionein quartile. Best viewed in MS-Excel.
(XLSX)

Table S3 **Gene-chemical interactions associated with sampling sites.** Best viewed in MS-Excel.
(XLSX)

Table S4 **Omic data altered with** ***Lepeophtheirus*** **infection.** Genes and metabolites altered with *Lepeophtheirus* infection in Morecambe Bay fish (T-test FDR<0.05). Best viewed in MS-Excel.
(XLSX)

Text S1 **Supplementary methods, results and discussion.**
(DOCX)
