# Supplementary figures and images for: Towards a System Level Understanding of Non-Model Organisms Sampled from the Environment: A Network Biology Approach
Source: PLoS Comput Biol. 2011 Aug 25;7(8):e1002126. doi: 10.1371/journal.pcbi.1002126 (PMC3161900; doi:10.1371/journal.pcbi.1002126)

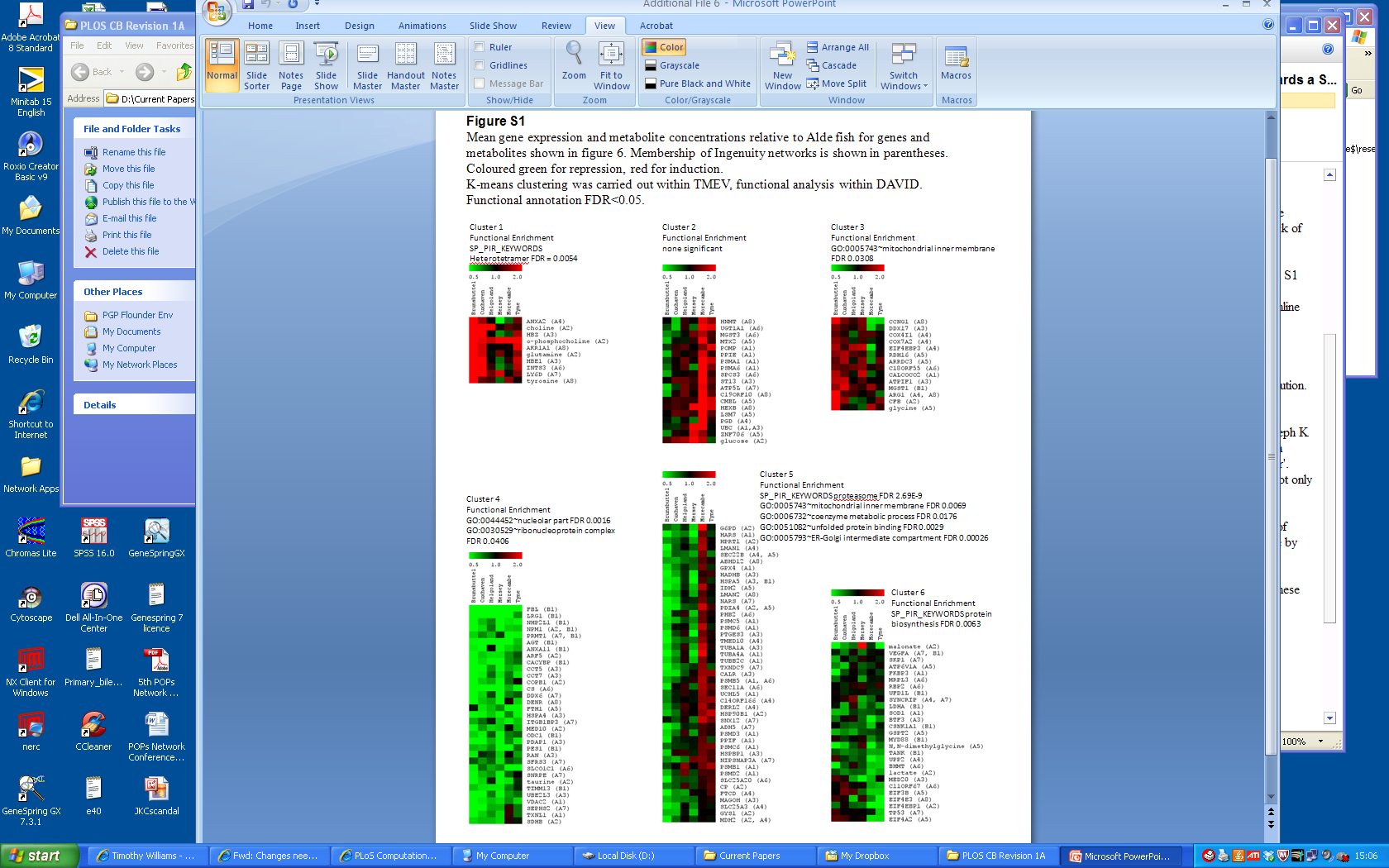

Supplement: Figure S1 — Expression of site-predictive genes and metabolites. Expression profiles of the site-predictive genes and metabolites shown in Figure 6, separated by K-means clustering and functionally annotated within DAVID. (DOCX) [file pcbi.1002126.s001.docx]
