# Supplementary material for: Towards a System Level Understanding of Non-Model Organisms Sampled from the Environment: A Network Biology Approach
Source: PLoS Comput Biol. 2011 Aug 25;7(8):e1002126. doi: 10.1371/journal.pcbi.1002126 (PMC3161900; doi:10.1371/journal.pcbi.1002126)
Supplement: Figure S2 — Modules overlapping with histopathology and chemical treatments. A to D – modules coloured red predict parasite infections and presence of histopathological liver abnormalities using GALGO with a sensitivity and specificity of >70%. E to L - modules coloured red significantly overlap (Fisher's Exact Test FDR<0.05) with transcripts significantly altering (ANOVA FDR<0.05) in response to laboratory exposures of flounders to individual stimuli over 16-day time courses. (PPTX) [file pcbi.1002126.s002.pptx]

## Slide 1
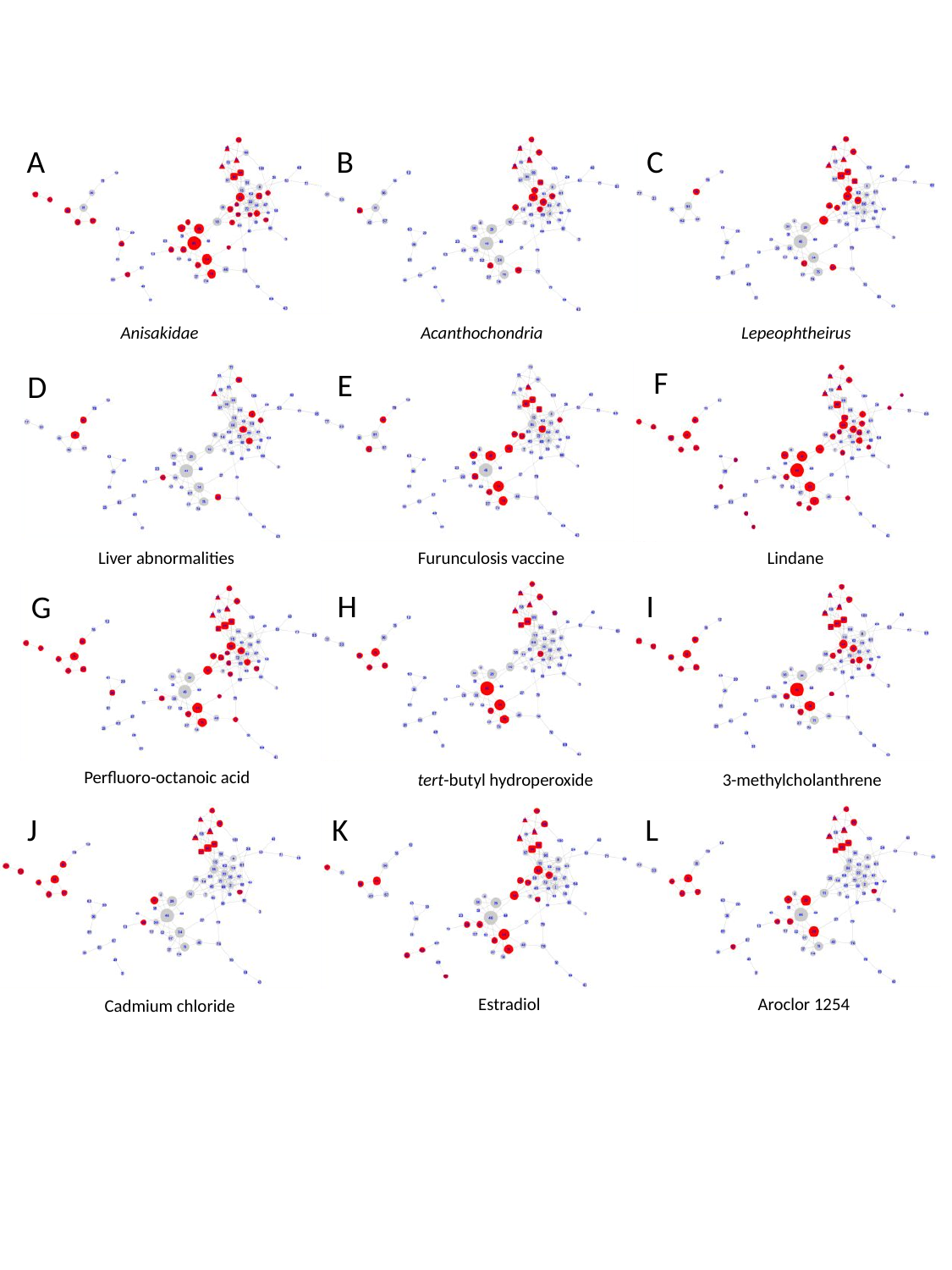

A
B
C
Anisakidae
Acanthochondria
Lepeophtheirus
F
E
D
Liver abnormalities
Furunculosis vaccine
Lindane
H
I
G
Perfluoro-octanoic acid
tert-butyl hydroperoxide
3-methylcholanthrene
J
K
L
Aroclor 1254
Estradiol
Cadmium chloride
