# Supplementary material for: Towards a System Level Understanding of Non-Model Organisms Sampled from the Environment: A Network Biology Approach
Source: PLoS Comput Biol. 2011 Aug 25;7(8):e1002126. doi: 10.1371/journal.pcbi.1002126 (PMC3161900; doi:10.1371/journal.pcbi.1002126)
Supplement: Figure S3 — Networks derived from modules that were predictive of sampling sites. A1 to A8 – Ingenuity networks derived from the union of modules that were highly predictive of sampling sites (5 or more), shown as major area A in Figure 3. B1 Ingenuity network derived from the module that was predictive of sampling sites (3), shown as minor area B in Figure 3. Ingenuity networks are coloured by mean gene expression in Brunsbuttel fish versus Alde fish with red for induction more than 2-fold, dark green for repression more than 2-fold, pink or light green for changes less than 2-fold. Uncoloured nodes were predicted by Ingenuity. (PPTX) [file pcbi.1002126.s003.pptx]

## Slide 1
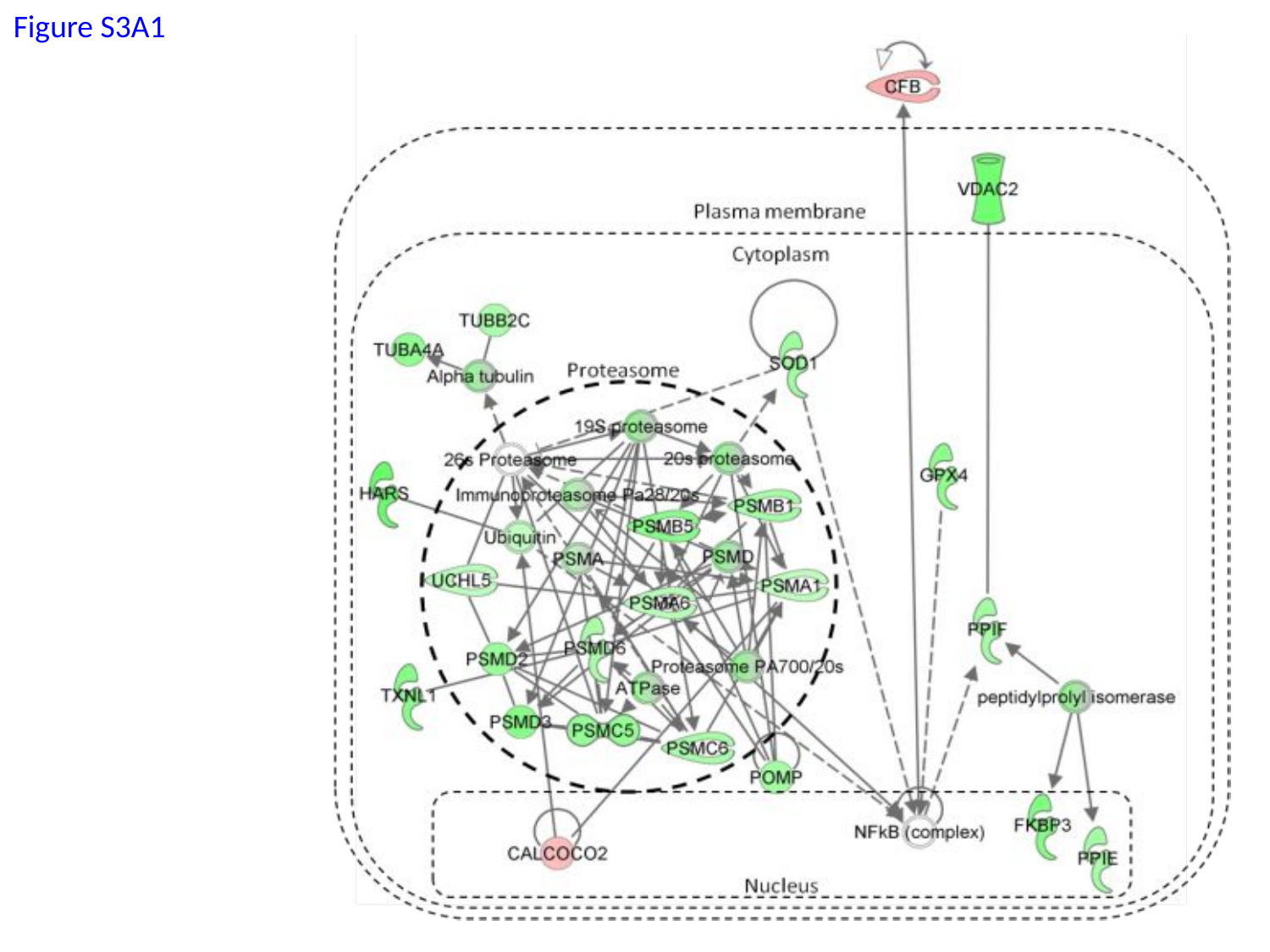

Figure S3A1

## Slide 2
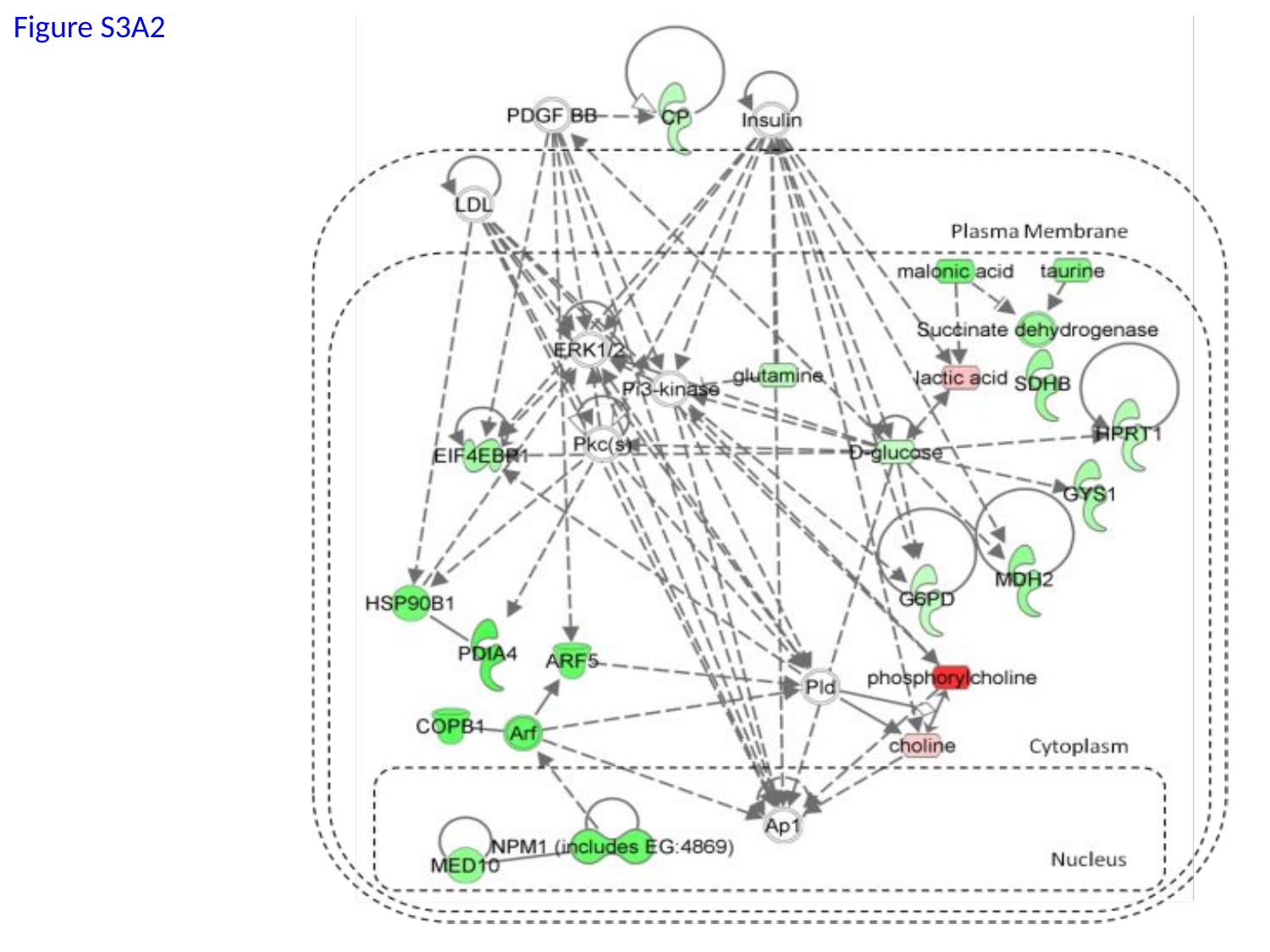

Figure S3A2

## Slide 3
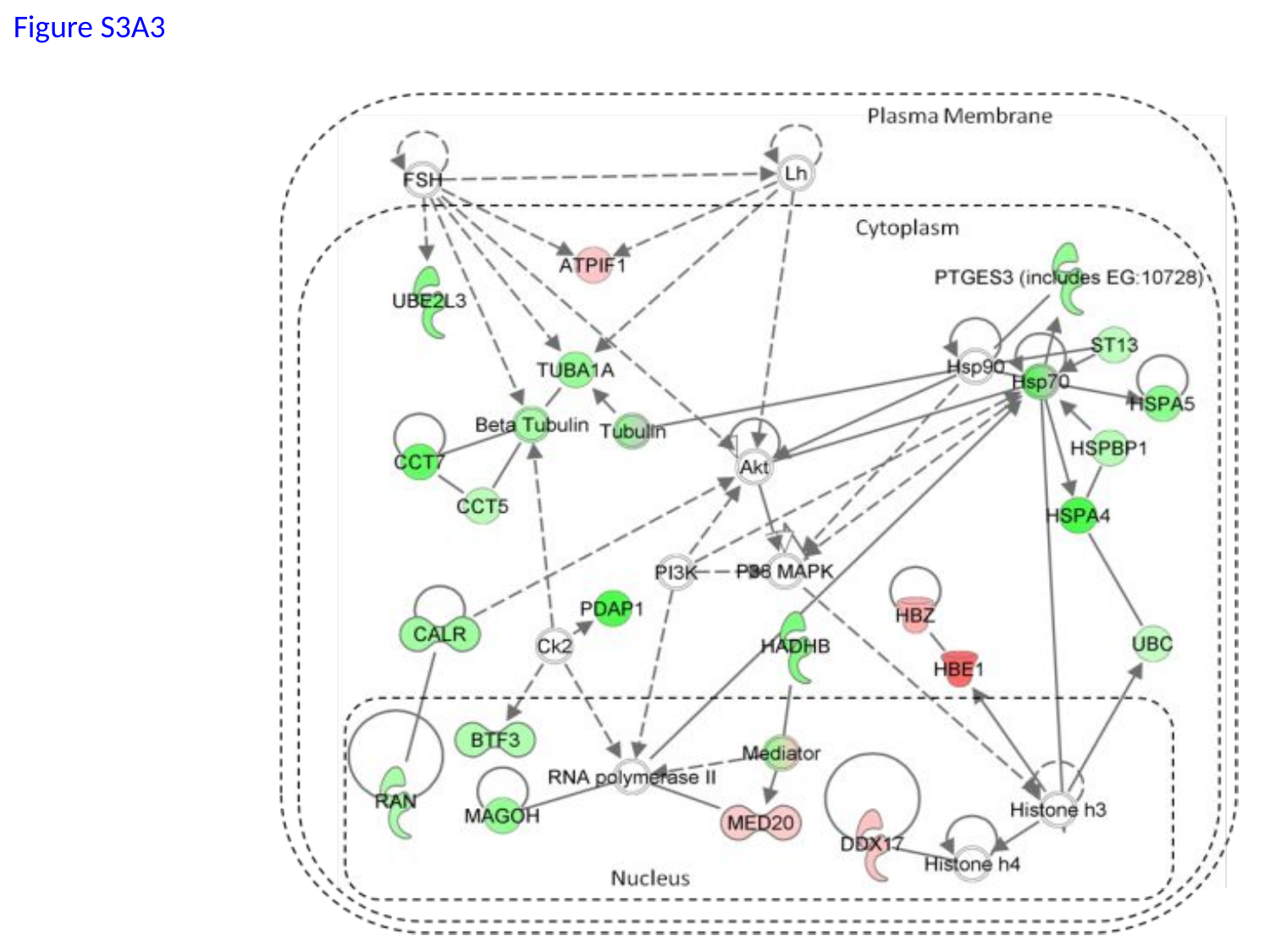

Figure S3A3

## Slide 4
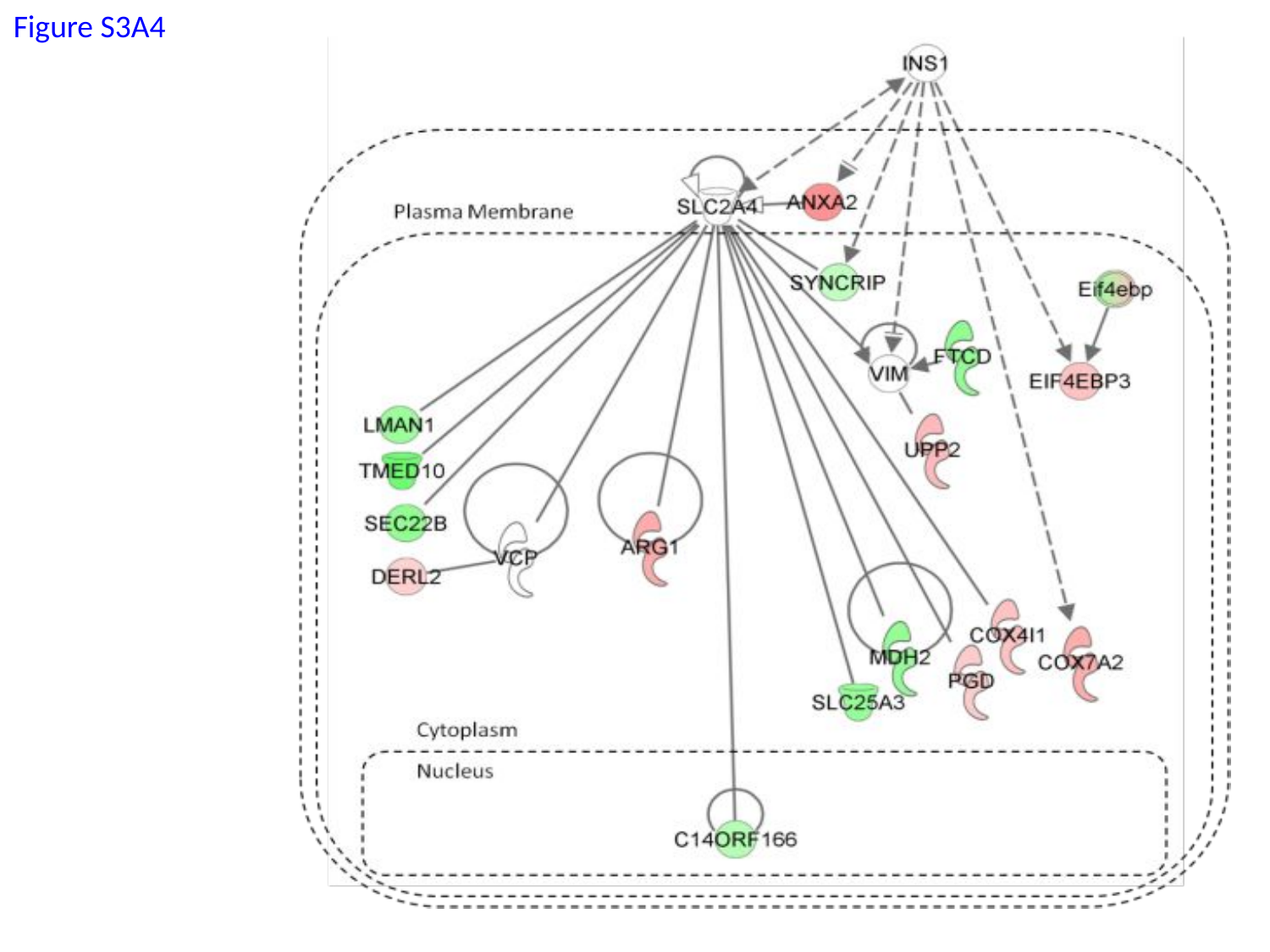

Figure S3A4

## Slide 5
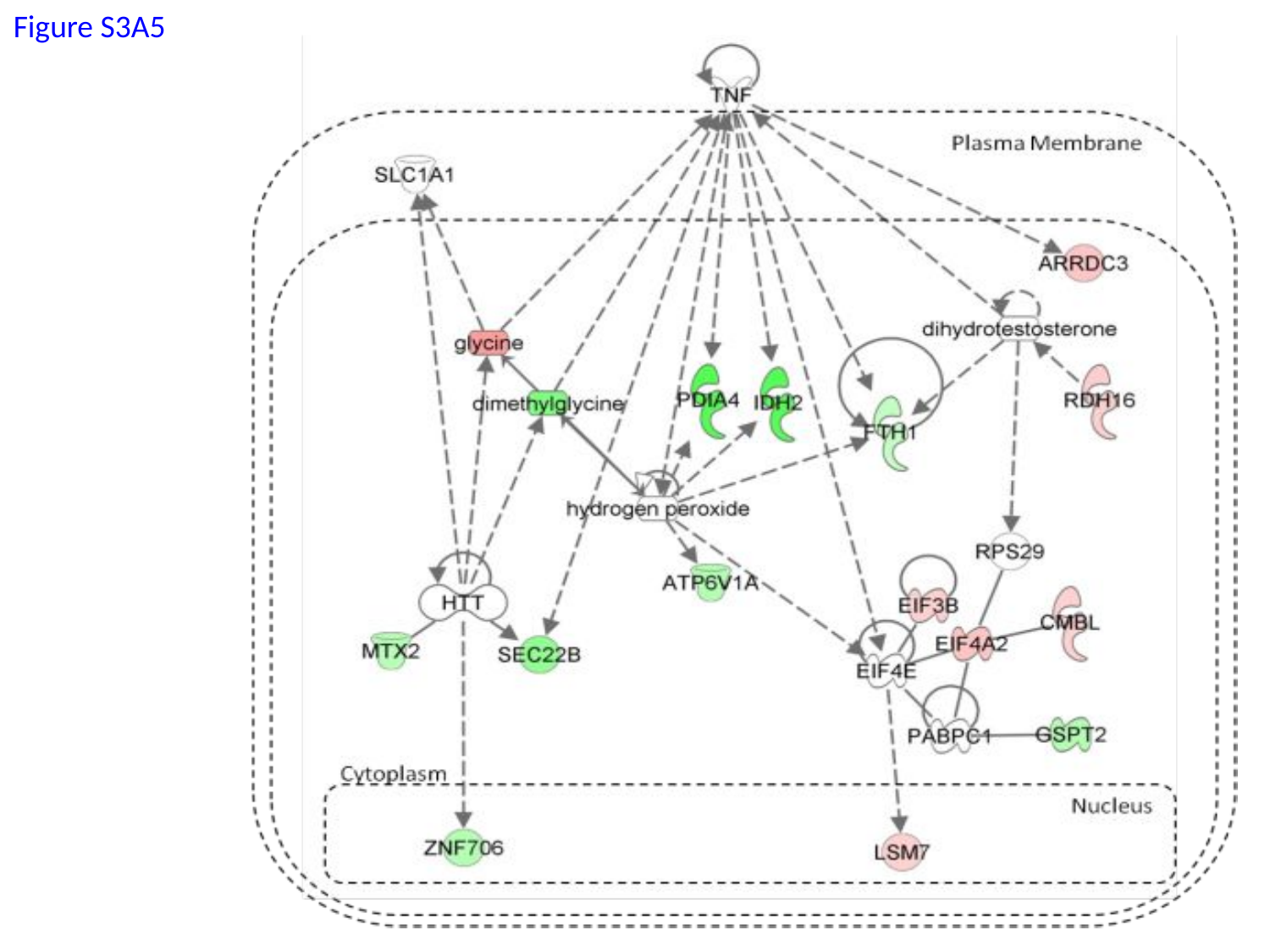

Figure S3A5

## Slide 6
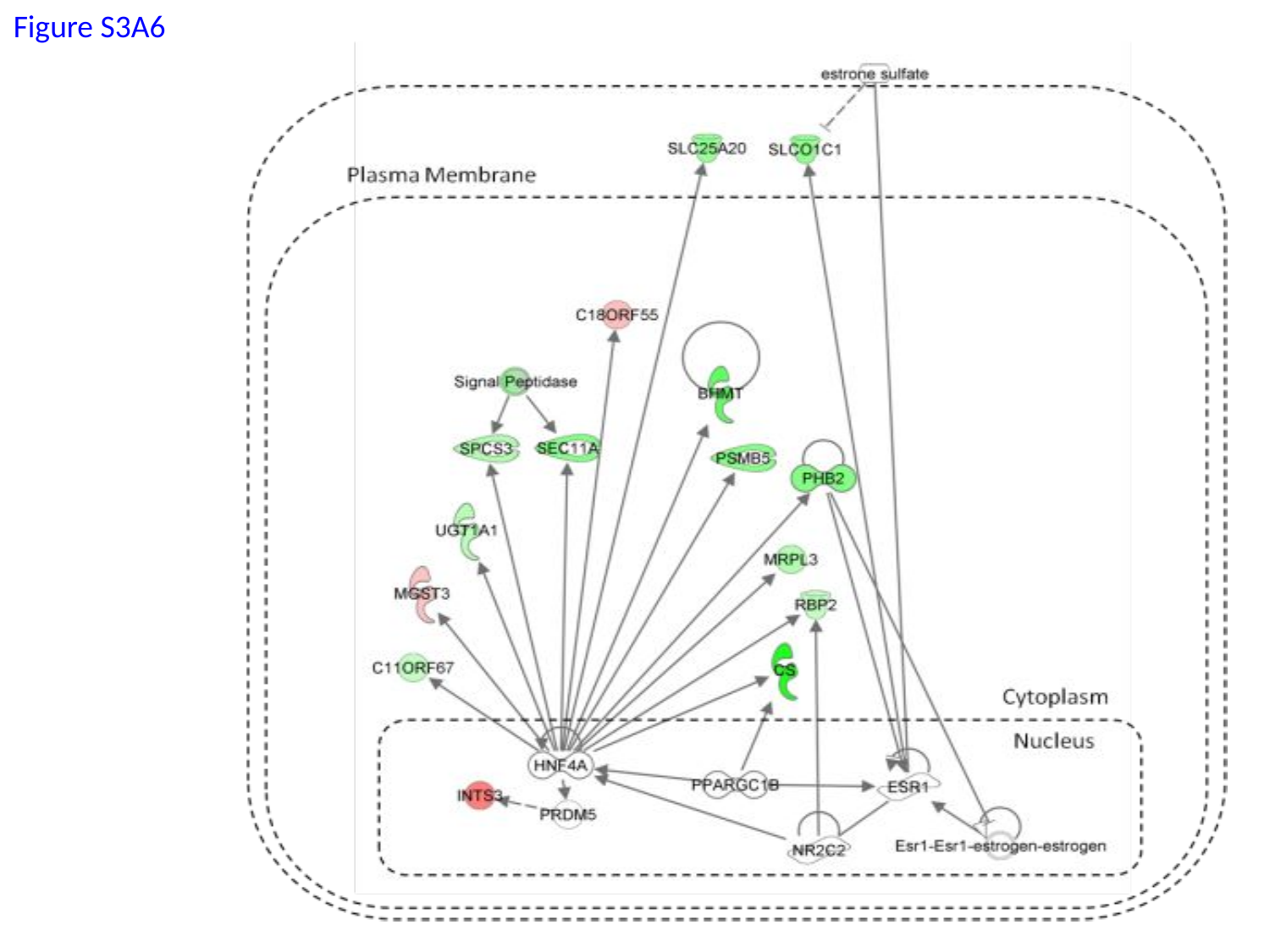

Figure S3A6

## Slide 7
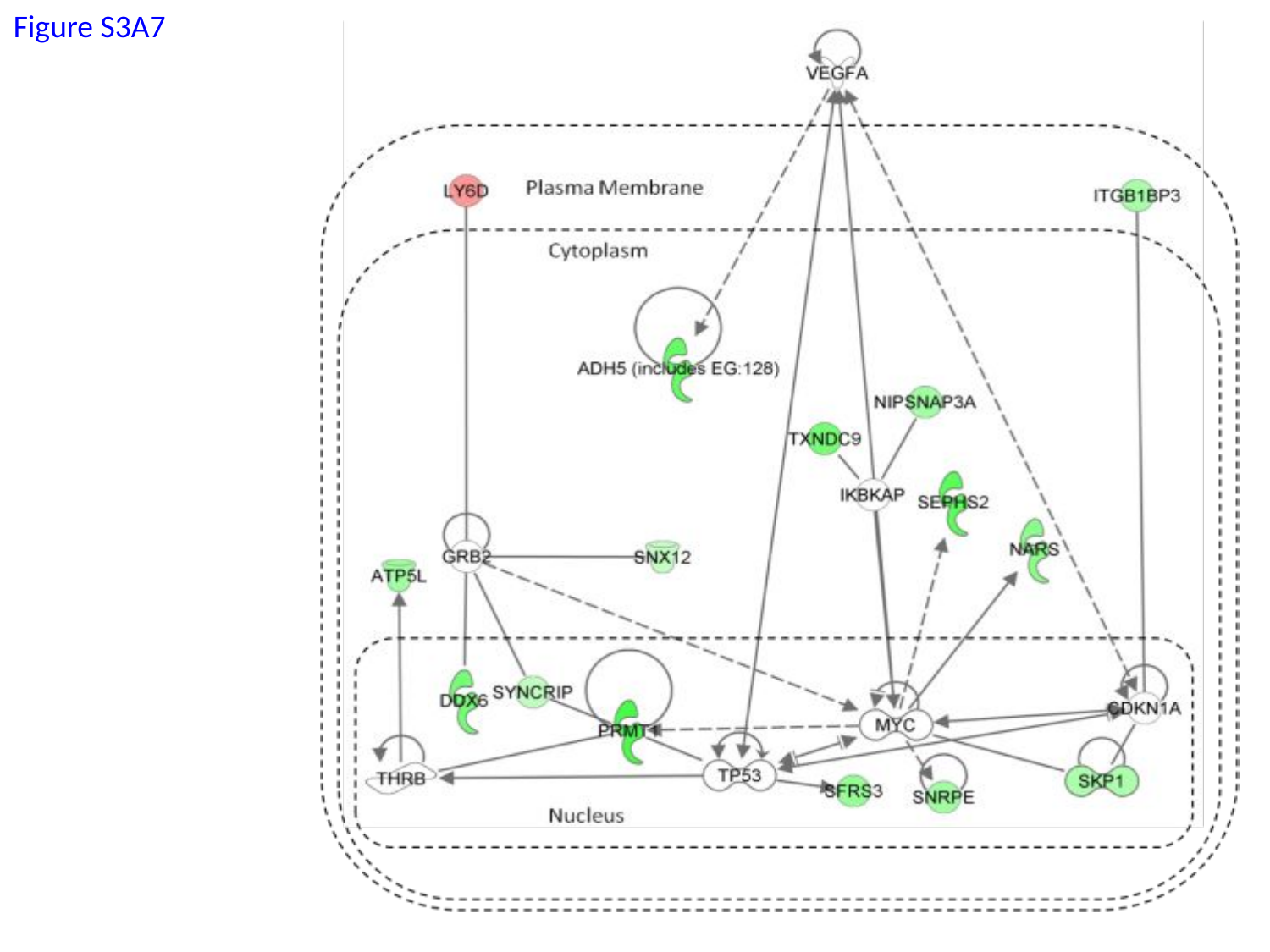

Figure S3A7

## Slide 8
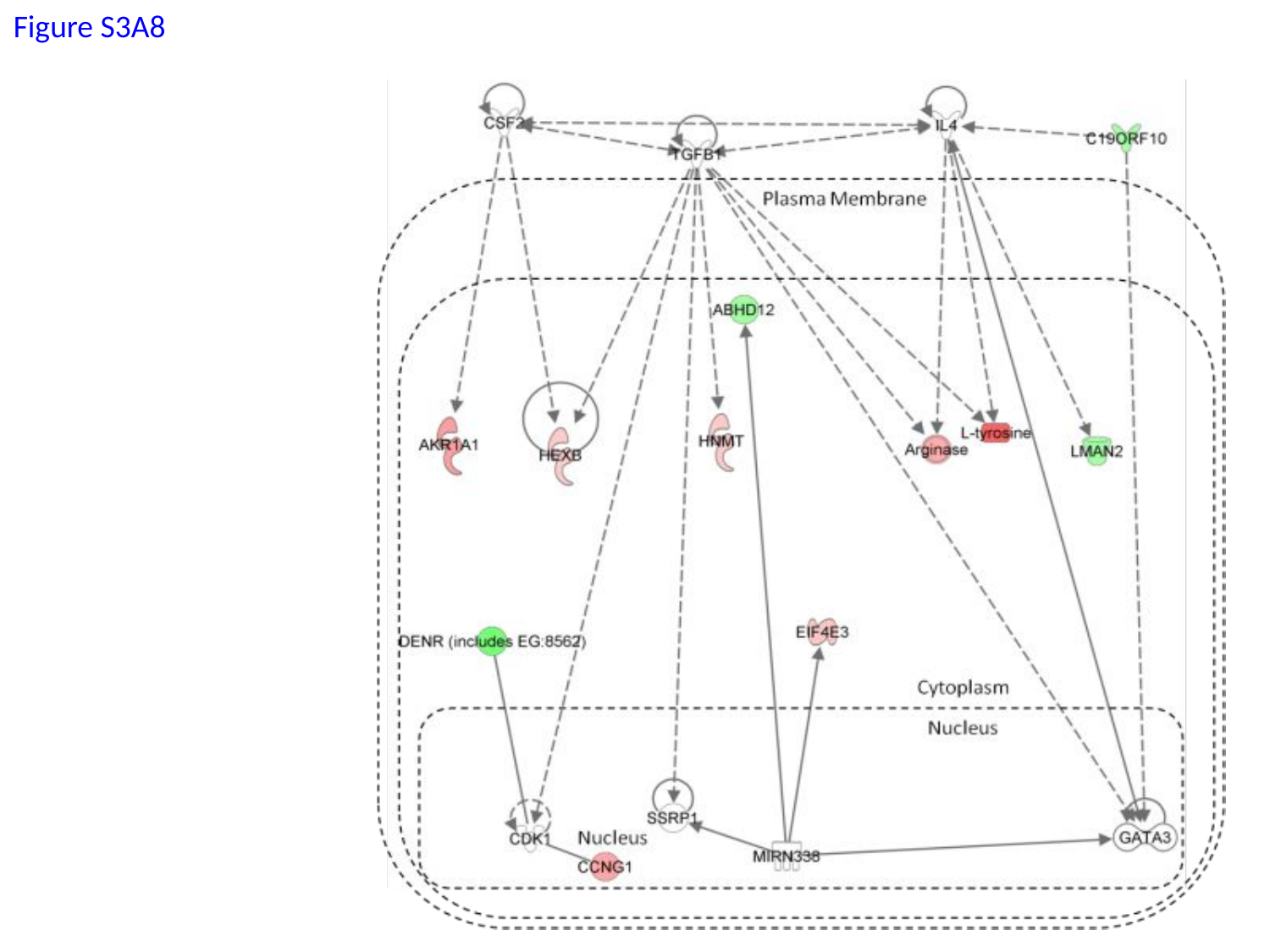

Figure S3A8

## Slide 9
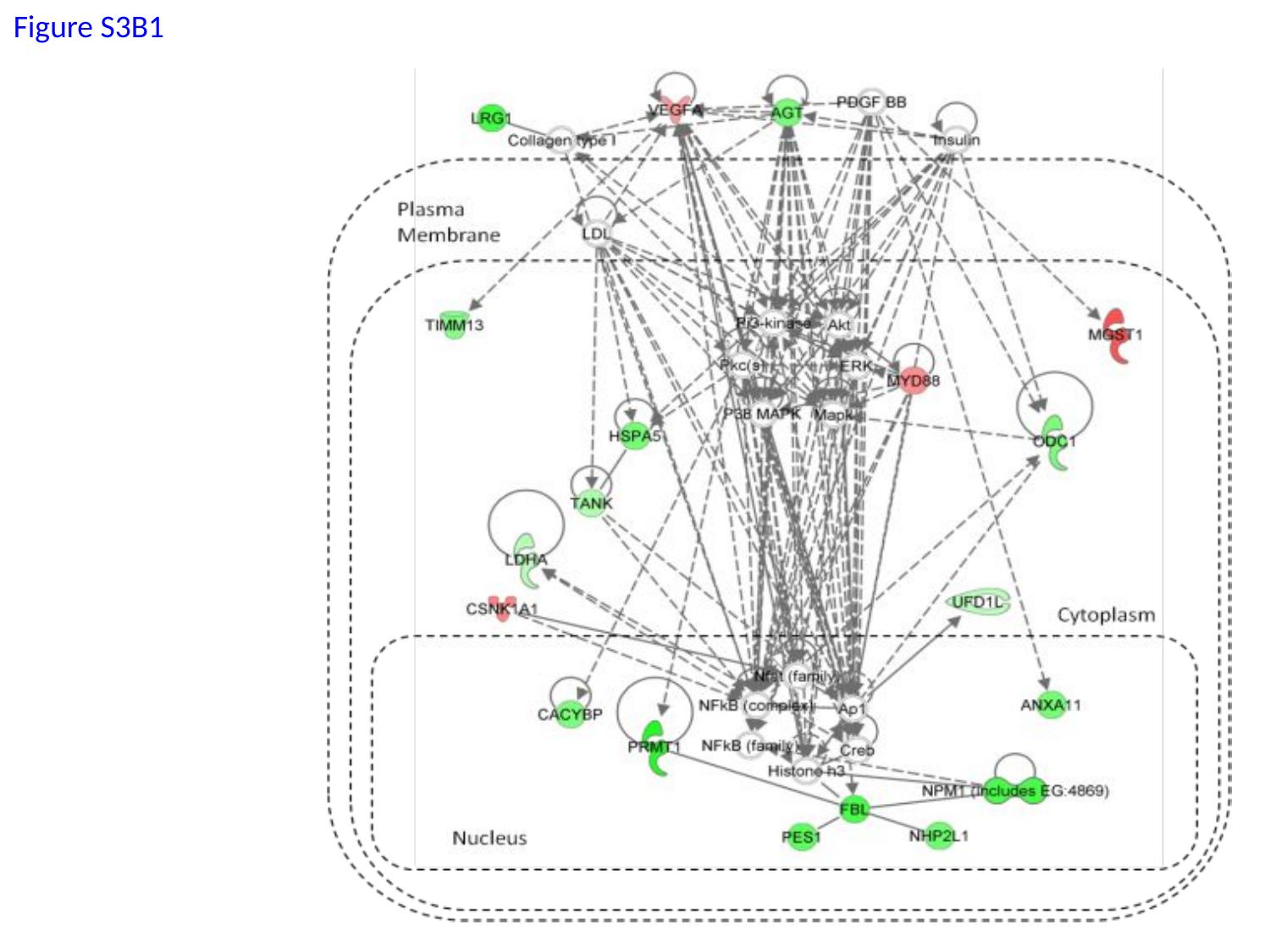

Figure S3B1
