# Supplementary material for: Towards a System Level Understanding of Non-Model Organisms Sampled from the Environment: A Network Biology Approach
Source: PLoS Comput Biol. 2011 Aug 25;7(8):e1002126. doi: 10.1371/journal.pcbi.1002126 (PMC3161900; doi:10.1371/journal.pcbi.1002126)
Supplement: Text S1 — Supplementary methods, results and discussion. (DOCX) [file pcbi.1002126.s008.docx]

# Text S1

**METHODS**

**Additional Network Inference Algorithm**

The additional algorithm for building clusters is very similar to one utilised in Abraham et al. [1] nonetheless the key ideas will be briefly discussed here. It is assumed that a symmetric matrix I with the same number of columns as the number of elements to be clustered is available. The off diagonal entries of this matrix are all positive and are the values of the mutual information values between the corresponding elements being clustered. In addition to the matrix a standardised clustering strength imin must be supplied by the user. The crucial first step in initializing the algorithm is to associate a node of an undirected graph with each element to be clustered and define edges between nodes when the mutual information exceeds imin. Thus the edge structure of the graph is determined by entries in the mutual information matrix and imin. The algorithm proceeds by executing a self avoiding random walk on the lattice defined by the graph where the starting point of the walk is a hub node. The second node visited is linked to the first, the third to the second and the first etc. and the algorithm halts when there are no new nodes to visit. The algorithm thus seeks to find cliques in the graph. The choice of which of the many possible nodes to visit at any stage is made by examining the number of neighbours of each candidate for inclusion and choosing one node using a coin flip biased towards nodes with a greater number of neighbours. The algorithm is thus based on a stochastic greedy heuristic.

In pseudo-code the algorithm is described below ( I(x,y) is an element of the Mutual Information matrix and ReturnSet is the output of the algorithm):

*G: undirected graph*

*S: set of all nodes in G*

*E: set of all edges in G ∀ S_i_ ,S_j_  ∈ S*

*∃ E_ij_ ∈ E I(G_i_,G_j_) > i_min_*

*ReturnSet ⊂ S: set of nodes in returned clique*

*CandSet ⊂ S*

*∅ ← CandSet*

*TempSet ⊂ S*

*∅ ← TempSet*

*S_j_ ← ReturnSet S_j_ ∈ is a hub node*

*CandSet ← {S_i_ ∈ S ∀S_i_ neighbour of S_j_ }*

*while CandSet ≠ ∅ do*

*∀S_i_ ∈ CandSet define n_i_ (number of neighbours) & p_i_ = (ni / ∑n_i_)*

*Select Sk ∈ CandSet with probability p_k_*

*Insert S_k_ in ReturnSet*

*TempSet ← {S_m_ ∈ S ∀S_m_ neighbor of S_k_ & S_m_ ⊄ ReturnSet}*

*CandSet ← (CandSet ∩ TempSet)*

*TempSet ← ∅*

*If CandSet ≡ ∅ Return ReturnSet*

The stochastic greedy aspect of the algorithm arises in the first two step of the while loop. Thus re-running the algorithm from the same starting point can give different values.

**RESULTS**

**Summary**

Adult male flounders sampled from seven environmental sites were analysed by morphometric parameters, liver histopathology, liver and serum protein and enzyme biomarkers, microsatellite genetic markers, hepatic transcriptomics and metabolomics, with chemical analyses carried out upon sediment samples and an independent cohort of flounder livers. The results obtained are shown in Table 1, S1 and S2. Briefly, fish sizes, weights and somatic indices differed between sites (Table 1A). The Tyne, Mersey and Brunsbuttel sites showed highest loads of xenobiotic pollutants, Cuxhaven and Helgoland intermediate levels and Morecambe and Alde relatively low levels, however there was considerable diversity in the representation of classes of toxicants, for example Tyne fish livers contained high levels of polycyclic aromatic hydrocarbons (PAHs) but relatively low levels of polychlorinated biphenyls (PCBs), whereas both were high in Brunsbuttel fish (Table 1 B to G). Histopathology of UK fish was consistent with these broad levels of pollution and copepod and nematode infection rates were highly site-associated (Table 1H). Serum vitellogenin (VTG) was modestly but significantly elevated in Brunsbuttel fish, hepatic glutathione reductase (GR), glutathione *S*-transferase (GST) and metallothionein (MT) were induced at various polluted sites with elevated heavy metal concentrations, ethoxyresorufin-*o*-deethylase (EROD) activity reflected both sediment and liver PAH concentrations (Tables 1I, S2). Microsatellite markers suggested a weak dichotomy between North Sea and Irish Sea fish. Microarray data are available from ArrayExpress at EMBL-EBI under accession E-MTAB-396. By ANOVA (FDR<0.05) 629 transcripts and 82 metabolite bins were significantly different between sites, of which 410 and 25 respectively were identifiable (Table S1). Within-site tests showed that morphometric parameters and parasite infections could be significantly associated only with a small proportion (<3%) of the gene expression differences between sites (Tables S1 and S4).

# a- Fish Sampling

Data describing the fish sampled are shown in Table 1A. All variables differed significantly between sampling sites. Brunsbuttel fish were the smallest, with lowest liver weight, gonad weight and gonadosomatic index (GSI), but high condition factor and hepatosomatic index (HSI). Mersey and Morecambe Bay fish were the largest. Condition factor was higher for the FRG-sampled fish than the UK-sampled fish, including for groups of comparable length such as Tyne and Helgoland, and was higher amongst Alde and Tyne fish compared with Mersey and Morecambe Bay fish.

**b- Chemistry**

Metal concentrations in sediment and an independent cohort of flounder livers are shown in Tables 1B and 1C respectively. All sites breached lower Oslo and Paris Commission (OSPAR) ecotoxicological assessment criteria (EAC) [2] for at least one metal, with all except Alde and Morecambe Bay breaching at least one upper EAC limit. Tyne exceeded upper EAC limits for Cd, Pb and Zn, Mersey and Brunsbuttel for Hg and Pb, Helgoland for Pb and Cuxhaven for Hg. While there is considerable diversity in the representation of different metals, an overview indicated that Tyne > Mersey & Brunsbuttel > Helgoland & Cuxhaven > Morecambe Bay > Alde in terms of metal concentrations. The partial data set for fish liver metals showed a comparable level of contamination in Alde, Tyne and Mersey fish and, where available, in FRG-sampled fish, although Mersey fish had a particularly high concentration of liver Hg (0.73 mg/kg wet wt.). There was little positive correlation between sediment and fish liver metal concentrations, the only cases with r^2^>0.5 were for Hg (r^2^=0.54) and Ni (r^2^=0.88), though the Ni correlation was based on only three points, therefore being of low significance. Interestingly there was an apparent inverse correlation for Cd (r^2^=0.70).

PAH concentrations in sediments and flounder livers are shown in Tables 1D and 1E respectively. Sediments at all sites except Alde and Morecambe Bay breached lower EAC limits for at least one PAH, with Brunsbuttel breaching one upper EAC limit, Tyne and Mersey breaching four and five respectively. Total PAH concentrations were Tyne > Mersey > Brunsbuttel > Helgoland & Cuxhaven > Morecambe Bay & Alde. Flounder liver PAH concentrations were available for Tyne, Mersey and Alde and followed the same pattern (r^2^>0.99, three points only).

PCB concentrations in sediments and livers are shown in Tables 1F and 1G respectively. Tyne, Brunsbuttel and Cuxhaven sediments exceeded lower EAC limits for the sum of ICES 7 PCBs, Mersey sediment exceeded the upper EAC limit. Sites were ordered by PCB contamination as Mersey > Tyne > Brunsbuttel > Cuxhaven > Helgoland > Alde & Morecambe Bay. In contrast, in fish liver, Brunsbuttel samples showed the highest PCB load, exceeding 1μg/kg wet wt. Sediment and fish liver PCB concentrations were not well-correlated. Fish liver samples were ordered Brunsbuttel > Cuxhaven > Mersey > Helgoland > Tyne & Morecambe Bay & Alde.

In broad terms, the Tyne, Mersey and Brunsbuttel showed high levels of contamination by pollutants, Cuxhaven and Helgoland intermediate levels and Morecambe Bay and Alde relatively low levels.

**c- Histopathology**

Histology and histopathology were carried out on UK samples only and data are shown in Table 1H. High numbers of fish were infected by *Lepeophtheirus* and *Acanthochondria* copepod parasites and *Anisakidae* nematodes, but there were relatively few infections by *Glugea* microsporidians. The occurrence of copepod infections and nematode infections appeared to be inversely related, copepod infections being particularly prevalent (>70%) at the less polluted Alde and Morecambe Bay sites, with nematode infections more prevalent at the more polluted Tyne and Mersey sites. Liver histopathology of the UK fish showed the highest occurrence of abnormalities in the Mersey samples, which included the only examples of hepatic tumour-bearing fish, one fish displayed hepatocellular adenoma and two hepatocellular carcionoma. Liver abnormalities were detected in 89% of Mersey fish, 75% of Tyne fish, 67% of Morecambe Bay fish and 40% of Alde fish. Liver histopathology was therefore consistent with broad pollution levels.

**d- Biomarker Assays**

Protein biomarker assays were performed on UK and Brunsbuttel fish, shown in Table 1I. All biomarkers significantly differed between sites by ANOVA (P<0.05), except VTG. Serum VTG concentrations were generally low, implying that these male fish were not exposed to high concentrations of xenoestrogens. However, VTG was modestly but significantly (single T-test P<0.05) elevated in Brunsbuttel fish and very highly induced (>1000-fold versus Alde) in one fish from the Mersey and one fish from Morecambe Bay. One fish from the Mersey and one fish from Morecambe Bay were also classified as intersex by histology, but these did not display elevated serum VTG concentrations. GR activity was lowest in Morecambe Bay fish and significantly higher in Tyne fish indicative of exposure to oxidative stressors [3] and indeed correlated well with liver Ni (r^2^=0.81), an acknowledged oxidative stressor [4] and moderately with sediment Cd, Zn and Pb (r^2^>0.6). Flounder liver GST activity is primarily attributable to two isoforms which detoxify lipid peroxidation products [5] and are inducible by organic peroxides and peroxisomal proliferators [6,7]. GST activity was lowest in Morecambe Bay fish. Alde, Tyne and Brunsbuttel fish showed significantly higher GST activity, though Alde was the least elevated (<2-fold) of these. GST correlated well with liver Cr and sediment As (r^2^>0.8) and moderately (r^2^>0.6) with sediment Ni, Pb and Zn and sediment and liver PAH (3 points only).

EROD, measuring CYP1 family enzyme activity, and inducible by AhR agonists including PAHs, PCBs and dioxin, was lowest in Alde samples, significantly higher in Mersey and Morecambe Bay fish and significantly elevated more than 3-fold in Brunsbuttel fish. Tyne fish showed an average 7-fold induction in EROD activity, but this was not statistically significant due to high inter-individual variability. EROD correlated highly with both sediment and liver PAH (r^2^>0.87), but using 3 points only. Morecambe Bay fish had the lowest hepatic MT concentration, and this was significantly elevated in Mersey and Brunsbuttel fish. Hepatic MT in flounder was induced moderately by Cd and Zn but not by Cu or Hg in a previous study [8]. MT correlated well with both sediment and liver Hg (r^2^>0.95) and moderately with liver Cr and sediment As (r^2^>0.6). Overall, the changes in protein biomarkers were classified as Tyne & Brunsbuttel > Mersey > Morecambe Bay & Alde. These reflected the degree of pollution revealed by the chemistry data, although the Mersey fish were less responsive than might be expected and fish from Morecambe Bay, which had relatively low sediment PAH and PCB concentrations, had significantly elevated EROD activity, possibly indicative of migration or an alternative exposure route.

**e- Genetics**

There was no evidence of significant departure from H/W expectations at any of the polymorphic loci. For the presumed neutral set of markers (microsatellites; mean 7.2 alleles per locus per sample) there were indications of a dichotomy between Irish Sea and North Sea samples, though this was, at best, weak. There was no evidence of population substructure within these two geographic areas. There was relatively little variability within the detoxification gene-associated genetic markers (mean 1.6 alleles per locus per sample) and nuclear transcription factor sequences (2.1 alleles per locus per sample) and neither robust geographic structuring nor grouping according to degree of pollution was discernible from these data.

**f- Transcriptomics and Metabolomics Data**

Microarray data, upon initial analysis, were found to be affected by slide print-batch bias; therefore, as samples had been randomized, a Bayesian correction was applied to resolve this problem. Subsequently, after finding representative clones for each contiguous sequence cluster and discarding unreliable data, 2417 non-contiguous transcripts passed the quality thresholds. For metabolomics, data from 560 discrete bins were carried through into further analyses. Transcriptomic and metabolomic data were combined, together with fish measurements length, weight, liver weight, HSI and K. ANOVA between samples grouped by site at FDR<0.05 showed that 629 transcripts, 82 metabolite bins and all fish measurements were statistically significantly different between sites. These data are shown in Table S1 for the transcripts that were identifiable with known protein coding sequences (410) and identified metabolite bins (25). Of particular note were those genes and metabolites that were consistently induced at all sites more than 1.5-fold in comparison with Alde fish, These included the metabolite o-phosphocholine and transcripts cytochrome P450 1A (CYP1A), the pro-apoptotic gene direct IAP binding protein with low pI (DIABLO) [9,10], ceruloplasmin, hepcidin and catalase. Of these, the well-known biomarker gene CYP1A was most highly induced. Overall, the gene expression changes showed similarities with, but were not identical to, the changes detected in our earlier study [11]. Gene ontology comparisons of differentially expressed genes showed changes in xenobiotic, glutathione and hormone metabolism, transcription, translation, ATP synthesis, cell cycle and metal ion homeostasis (Text S1, Figure 1).

Since fish measurements were significantly different between sites, T-tests at FDR<0.05 were carried out within each site group comparing the top and bottom quartile for each parameter; length, weight, liver weight, gonad weight, HSI, GSI and K. One glucose bin (3.482) was associated with GSI, phospholipase A2 and an EST weakly similar to 14kDa apolipoprotein were associated with liver weight, N,N-dimethylglycine, six unidentified metabolites, UDP-glucuronosyltransferase (UGT3 gene), serine hydroxymethyl transferase 1 (SHMT1), chemokine (C-C motif) ligand 14 and betaine aldehyde dehydrogenase were associated with condition factor (K). Of these, glucose, UGT3 and SHMT1 were identified as differentially expressed between sites (Table S1). Similarly, as parasite occurrence was different between sites, T-tests (FDR<0.05) were carried out within each UK site group comparing fish with and without *Lepeophtheirus*, *Acanthochondria*, *Anisakidae* and *Glugea* infections. Only *Lepeophtheirus* infection resulted in statistically significant differences in gene expression and metabolite concentration, with 3 unidentified ESTs differentially expressed in Alde fish, and 15 metabolite bins and 60 transcripts altered in Morecambe Bay fish (Table S4). Significantly induced genes included Mx1, Mapk12, transferrin and STAT1, and were significantly associated with liver proliferation FDR=4.22E-3 and non-significantly with interleukin 22 and interferon signalling (Ingenuity). Nine of the identified genes were also differently expressed between sampling sites (Tables S1 and S4). Other comparisons identified no significant changes.

To compare the current data with previous transcriptomic data on independent samples from Alde, Brunsbuttel, Helgoland, Cuxhaven and Tyne sites Howdon and Team [11], a classification algorithm was employed and this predicted all sampling sites correctly, in agreement with the previous data and analysis, except for Cuxhaven that was assigned to Alde.

Blast2GO analyses (Text S1, Figure 1) showed increased representation of GO terms related to hormone metabolism in Brunsbuttel, xenobiotic and glutathione metabolism and ATP synthesis in Morecambe Bay and metal ion homeostasis in Tyne, Cuxhaven and Morecambe Bay. Cell cycle was over-represented amongst repressed transcripts at Helgoland, as were ER, ATP synthesis and metal ion homeostasis at Brunsbuttel. A number of terms related to transcription and translation were less common than expected amongst induced transcripts at the FRG sites and Mersey.

Genes and metabolites that were significantly different between sites and whose means by site positively correlated with sediment chemistry and fish liver chemistry and those passing T-tests at FDR<0.05 based on histopathlogy and biomarker quartile comparisons are shown in Table S2. Notably, CYP1A was highly correlated (r>0.835) with sediment PAH concentration, but also correlated with Cd, Cu, Hg and Zn. Ceruloplasmin was correlated with Hg and metabolites glutamine, malonate, glucose and N,N-dimethylglycine with PCB. Glutamine correlated with fish liver PCBs, as did cyclin G1, AKR1A1, haemoglobins and microsomal GST1 amongst others. Functions significantly enriched amongst genes and metabolites based on histopathology are shown in Table 3. These include associations between phospholipidosis and liver cholestasis and steatohepatitis, oxidative stress and xenobiotic metabolism, intersex with oxidative stress and mitochondrial dysfunction, vacuolar foci of cellular adhesion (FCA) with the acute phase response.

**g- Description of Ingenuity Networks**

The most significant network generated by Ingenuity from group A, (Figures 6A and S3A1) showed down-regulation of proteasomal subunit transcripts in Brunsbuttel fish, linked with a downregulation of proteasome maturation protein (POMP). In the network, the modulation of the proteosomal complex is directly linked to the nuclear factor kappa B (NF kappa B) transcriptional complex.

Figure S3A2 represents several gene-metabolite interactions related to energy pathways (glucose, lactic and malonic acid linked to succinate dehydrogenase and glucose-6-phosphate dehydrogenase) and phospholipid biosynthesis (choline, phosphocholine). The expression of energy-related enzymes and metabolites is linked to insulin, low-density lipoprotein, a protein kinase cascade consisting of extracellular signal-related kinase (ERK1) and protein kinase C and the immediate-early stress response factor Ap1. More specifically, the Ap1 response appeared to be mediated by nucleophosmin via ADP-ribosylation factor (Arf). Figure S3A3 represented a partially endocrine-driven response, with follicle stimulating hormone and luteinizing hormone inferred to act via protein kinases upon RNA polymerase II and associated transcriptional mediator proteins. These were linked with repression of heat shock proteins and tubulins. Figure S3A4 again highlighted implied effects of insulin, either independently or via the glucose transporter SLC2A4. Genes affected included those encoding protein trafficking molecules (SEC22B, TMED10) and mitochondrial cytochrome oxidases (COX4I1, 7A2). Figure S3A5 illustrated an interaction between the pro-inflammatory tumour necrosis factor (TNF) and oxidative stress (peroxide). Both metabolites (glycine, dimethylglycine) and transcripts (eukaryotic translation initiation factors) were affected and there was potential interaction with androgens, with the inferred presence of dihydrotestosterone within the network. Figure S3A6 showed a number of hepatocyte nuclear factor 4A (HNF4A) –responsive genes, with nuclear interactions between HNF4A and estrogen receptor alpha (ESR1), and a potential link to circulating levels of estrogen sulphate. Figure S3A7 suggested an interaction between vascular endothelial growth factor A (VEGFA) and key nuclear regulators of the cell cycle, DNA damage response and oncogenesis, including TP53, MYC and CDKN1A. Figure S3A8 showed responses linked to the cytokine interleukin 4 (IL4) and transforming growth factor beta 1 (TGFB1) , affecting cytosolic target genes and metabolites and again linking to cell cycle and DNA-damage response -related genes such as cyclin dependent kinase 1 (CDK1) and structure specific recognition protein 1 (SSRP1).

Figure 6B (Figure S3B1) shows the most significant network generated from module 40, group B of the site-predictive modules. This showed a complex picture involving a highly interlinked cytosolic protein kinase cascade linked to nuclear transcription factors including Nf kappa B, Ap1 and Creb. The extracellular growth factor VEGFA was induced, angiotensinogen was transcriptionally repressed, and platelet derived growth factor (PDGFBB), insulin and LDL were also predicted to be components of this response.

**DISCUSSION**

Both sediment and fish liver chemical analyses demonstrated that pollutant concentrations exceeded statutory limits, especially at the Tyne, Mersey and Brunsbuttel locations. This showed that anthropogenic pollution of the aquatic environment was still of high concern in Western European waters notwithstanding the major advances in regulation and water treatment. There was a lack of correlation between concentrations of metals and PCBs measured in sediment and fish livers. To some extent this may be due to differences in metabolism and excretion, but there is a likelihood that variations in point sources of contamination within an estuary could lead both to specific sediment samples being un-representative of the area, and to high variation in chemical exposure and responses in fish dependent on their recent and historical locations within the estuary. Due to the lack of chemical measurements for individual fish (which may have been un-representative in many cases due to the lack of connection between fish liver and sediment chemistry), individual correlations were not possible. A number of genes and metabolites correlated with average concentrations of different toxicants, but these are likely to represent non-specific responses to pollutants, due to the similar profiles for various toxicants between different sites (Tables 1,S2).

Liver abnormalities discovered by histopathology proved to be a useful way of classifying flounder sampling sites by broad pollution impact, as has previously been shown [12]. When examined by within-site comparisons, parasite infections were found to alter the expression of a limited set of genes and metabolites (Table S4), nine of which overlapped with identified genes that were differentially expressed between sites (Table S1). Parasites that are carcinogenic to humans, such as liver flukes, have been described in fish [13] and parasitic infections of fish can alter oxidative stress parameters [14], while conversely chemical contaminants can suppress immune system function [15]. Previous studies in flatfish have not demonstrated correlations between tumour incidence and parasite burden [12,15]. The transcripts that altered with *Lepeophtheirus* infection (Table S4) do suggest some interaction with mechanisms of carcinogenesis. For example a cyclin-dependent kinase and MAP kinase were induced while paraoxonase and a cytochrome P450 family 2 gene were repressed in infected fish at Morecambe Bay. However, these expression changes were not detected in response to infection at any other site. From these results the parasite-associated molecular changes are a minor, but important, component of the flounder hepatic molecular differences between sites. While fish morphological measurements differed between sites, examining the within-site differences related to these factors revealed only modest effects upon gene expression and metabolite concentrations in the liver, therefore implying that the majority of the omic differences between sites were not due to differences in the sizes of the fish.

Biological effects of planar PAH-type compounds (PAHs, PCBs and dioxins), heavy metals (Cd, Cu and Zn), oxidative stressors and estrogenic compounds were assessed by assays of biomarker phenotypes (EROD, MT, GST, GR, VTG). These showed measurable differences and positive responses in fish from all sampling sites except for estrogenic effects which were confined to fish from the two Irish Sea environments (Morecambe Bay and Mersey) and Brunsbuttel. Severe impacts of PAH–type compounds, measured by EROD, were observed in 20% of Tyne fish and 40% of Brunsbuttel fish, for heavy metals, measured by MT, in 12% of Mersey fish and 40% of Brunsbuttel fish and for oxidative stressors, measured by GR, in 5% of Alde and 25% of Tyne fish. Whilst these different phenotypes could be roughly equated with the differences in the chemical contaminants present in the sampling locations there were anomalies that might be explained by differences in the residence times of individual fish within the sampling areas. Although biomarker responses were correlated with certain chemical contaminants, few associations were found with omic data, except for hepatic metallothionein protein (Table S2).

The main finding of the genetic study was a weak dichotomy between North Sea and Irish Sea fish. The neutral microsatellite, locus 3F10, was the only marker significantly linked to gene and metabolite expression changes. Based upon these data we conclude that population genetic differences do not have a major influence on the measured phenotypic variables in these flounder.

*Individual genes and metabolites*

Examples of individual genes generally highly induced at the polluted sites included CYP1A, DIABLO, hepcidin, catalase, and transthyretin (Table S1). We have previously found these and many other of the identified genes to be altered in expression at the polluted sites [11], indeed CYP1A [16] and catalase are well-known biomarkers of planar aromatic contaminants and oxidative stress, respectively. Based upon this induction of CYP1A it was likely that the AhR pathway was activated at all sites in comparison with Alde. Aldehyde dehydrogenase ALDH9A1, microsomal GSTs and Nrf2 target genes of the oxidative stress response such as catalase, thioredoxin and aldo-keto reductase AKR1A1 were also induced at polluted sites. Additional highly responsive genes included DIABLO and haemoglobins. Based upon interaction networks (Figure 7) we hypothesise that induction of DIABLO is AhR dependent in flounder. In mammals DIABLO is positively regulated by BAX [17], an AhR responsive factor [18]. The induction of pro-apoptotic DIABLO, previously found induced in flounder hepatocytes from fish chronically exposed to polluted sediments [10], negatively affects the anti-apoptotic gene survivin (BIRC5) [19], leading to an increase in caspase 3 dependent apoptosis. We indeed found an apparent mild repression of survivin and induction of caspase 3. Haemoglobins can be regulated by AhR-induced CDKN1B [20,21], alternatively, the apparent induction of haemoglobin mRNA may be related to infiltration of erythrocytes into damaged liver tissue.

The metabolite o-phosphocholine was induced at all sites and choline was induced at Brunsbuttel and Morecambe Bay, in comparison with Alde fish (Table S1). These molecules are intermediates in phosphatidylcholine synthesis; therefore these alterations imply changes in phospholipid concentrations. At the polluted sites, fish were more likely to suffer from phospholipidosis (Table 1H), a lysosomal storage disorder where excess phospholipid accumulation occurs. Therefore choline and o-phosphocholine may represent useful metabolic biomarkers for this condition, indeed, choline concentration was significantly associated with phospholipidosis (Table S2). Previously we have investigated hepatic differences between female flounders from the Tyne and Alde estuaries by metabolomics [22] and discovered repression of lactate and glucose at the Tyne site. In the current experiment lactate was also repressed in male fish from the Tyne, but glucose was not consistently affected (Table S1).

While focussing on individual genes and metabolites is undoubtedly informative, it was apparent that the many hundreds of significant changes identified required higher level analyses, to predict both potential exposures and health outcomes and to focus on responsive pathways. These analyses are shown and discussed within the main body of this publication.

**REFERENCES**

1. Abraham K, Sameith, K, Falciani, F: **Improving functional module detection**. In: *Ohio Collaborative Conference on Bioinformatics: 2009; Ohio*; 2009: 110-115.

2. OSPAR: **OSPAR/ICES Workshop on the evaluation and update of background reference concentrations (B/RCs) and ecotoxicological assessment criteria (EACs) and how these assessment tools should be used in assessing contaminants in water, sediment and biota.** Final Report. The Hague: OSPAR; 2004.

3. Sabine V, Minghetti M, Diab A, George S: **Glutathione reductase probes as a marker for oxidative stress**. *Mar. Environ. Res.* 2006, **62** Suppl.S452

4. Valko M, Rhodes CJ, Moncol J, Izakovic M, Mazur M: **Free radicals, metals and antioxidants in oxidative stress-induced cancer**. *Chem Biol Interact* 2006, **160**(1):1-40.

5. Leaver MJ, Wright J, George SG: **Structure and expression of a cluster of glutathione *S*-transferase genes from a marine fish, the plaice (*Pleuronectes platessa*)**. *Biochemical Journal* 1997, **321**:405-412.

6. Scott K, Leaver M, George SG: **Regulation of glutathione *S*-transferase A expression in flounders**. *Mar. Environ. Res.* **34**:233-236

7. Martinez-Lara E, Leaver M, George S: **Evidence from heterologous expression of glutathione *S*-transferases A and A1 of the plaice (*Pleuronectes platessa*) that their endogenous role is in detoxification of lipid peroxidation products**. *Mar Environ Res* 2002, **54**(3-5):263-266.

8. George S, Wright J, Carpene E, Kindt M: **Kinetics and magnitude of metallothionein induction by Cd, Cu, Hg and Zn in European flounder - calibration for environmental monitoring.** *Mar. Environ. Res* 1999, **50**:433-434

9. Williams TD, Diab A, Ortega F, Sabine VS, Godfrey RE, Falciani F, Chipman JK, George SG: **Transcriptomic responses of European flounder (*Platichthys flesus*) to model toxicants**. *Aquat Toxicol* 2008, **90**(2):83-91.

10. Leaver MJ, Diab A, Boukouvala E, Williams TD, Chipman JK, Moffat CF, Robinson CD, George SG: **Hepatic gene expression in flounder chronically exposed to multiply polluted estuarine sediment: Absence of classical exposure 'biomarker' signals and induction of inflammatory, innate immune and apoptotic pathways**. *Aquat Toxicol* 2010, **96**(3):234-245.

11. Falciani F, Diab AM, Sabine V, Williams TD, Ortega F, George SG, Chipman JK: **Hepatic transcriptomic profiles of European flounder (*Platichthys flesus*) from field sites and computational approaches to predict site from stress gene responses following exposure to model toxicants**. *Aquat Toxicol* 2008, **90**(2):92-101.

12. Stentiford G, Bignell, JP, Lyons, BP, Feist, SW: **Site-specific disease profiles in fish and their use in environmental monitoring**. *Marine Ecology Progress Series* 2009, **381**:1-5.

13. Smout MJ, Sripa B, Laha T, Mulvenna J, Gasser RB, Young ND, Bethony PJ, Loukas A: **Infection with the carcinogenic human liver fluke *Opisthorchis viverrini*.** *Mol Biosyst* 2011, DOI: 10.1039/c0mb00295j

14. Garcia LO, Becker AG, Bertuzzi T, Cunha MA, Kochhann D, Finamor IA, Riffel APK, Llesuy S, Pavanato MA, Baldisserotto B: **Oxidative stress parameters in silver catfish (Rhamdia quelen) juveniles infectedwith *Ichthyophthirius multifiliis* and maintained at different levels of water pH.** *Veterinary Parasitology* 2011, In Press doi:10.1016/j.vetpar.2010.12.039.

15. Stentiford GD, Bignell JP, Lyons BP, Thain JE, Feist SW: **Effect of age on liver pathology and other diseases in flatfish: inplications for assessment of marine ecological health status**. *Marine Ecology Progress Series* 2010, **411**:215-230.

16. Kirby MF, Smith AJ, Rooke J, Neall P, Scott AP, Katsiadaki I: **Ethoxyresorufin-O-deethylase (EROD) and vitellogenin (VTG) in flounder (*Platichthys flesus*): system interaction, crosstalk and implications for monitoring**. *Aquat Toxicol* 2007, **81**(3):233-244.

17. Henry H, Thomas A, Shen Y, White E: **Regulation of the mitochondrial checkpoint in p53-mediated apoptosis confers resistance to cell death**. *Oncogene* 2002, **21**(5):748-760.

18. Matikainen T, Perez GI, Jurisicova A, Pru JK, Schlezinger JJ, Ryu HY, Laine J, Sakai T, Korsmeyer SJ, Casper RF *et al*: **Aromatic hydrocarbon receptor-driven Bax gene expression is required for premature ovarian failure caused by biohazardous environmental chemicals**. *Nat Genet* 2001, **28**(4):355-360.

19. Verhagen AM, Vaux DL: **Cell death regulation by the mammalian IAP antagonist Diablo/Smac**. *Apoptosis* 2002, **7**(2):163-166

20. Munoz-Alonso MJ, Acosta JC, Richard C, Delgado MD, Sedivy J, Leon J: **p21Cip1 and p27Kip1 induce distinct cell cycle effects and differentiation programs in myeloid leukemia cells**. *J Biol Chem* 2005, **280**(18):18120-18129.

21. Marlowe JL, Puga A: **Aryl hydrocarbon receptor, cell cycle regulation, toxicity, and**

**tumorigenesis**. *J Cell Biochem* 2005, **96**(6):1174-1184.

22. Viant MR, Bearden DW, Bundy JG, Burton IW, Collette TW, Ekman DR, Ezernieks V, Karakach TK, Lin CY, Rochfort S *et al*: **International NMR-based environmental metabolomics intercomparison exercise**. *Environ Sci Technol* 2009, **43**(1):219-225.

**Text S1, Figure 1**


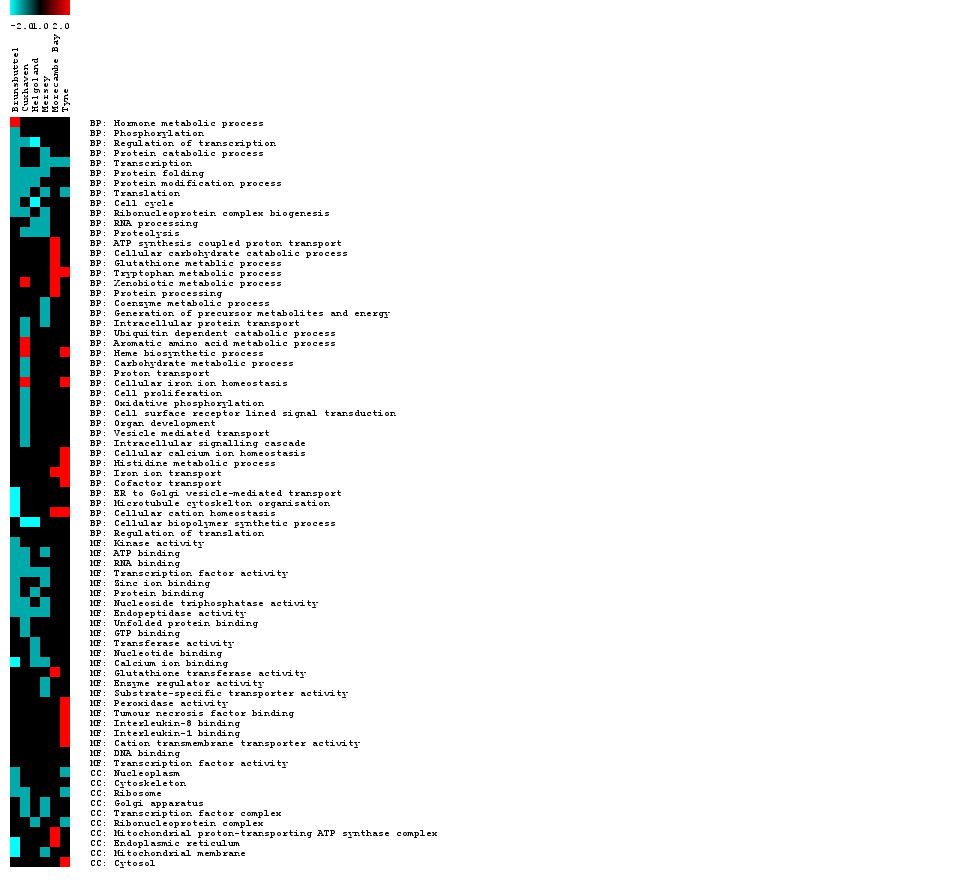


Heatmap displaying GO terms (most specific only) significantly enriched (FDR<0.05) between transcripts significantly differentially expressed between site (FDR<0.05) and 1.5-fold induced or repressed versus Alde and all detectable transcripts. Red denotes GO terms enriched amongst induced transcripts, bright blue GO terms enriched amongst repressed transcripts and dull blue GO terms less common than expected amongst induced transcripts, black indicates no enrichment.
